# Packaging Metal Atomic Clusters for Universal and Robust Protection through Cluster Beam Process

**DOI:** 10.1002/advs.202503347

**Published:** 2025-04-03

**Authors:** Siqi Lu, Zixiang Zhao, Jinsen Han, Jun Wang, Yongxin Zhang, Yu Du, Fangyu Guo, Zhichao Wang, Shenghui Wang, Sichen Tang, Kuojuei Hu, Jianguo Wan, Jiayu Dai, Fengqi Song

**Affiliations:** ^1^ National Laboratory of Solid State Microstructures Collaborative Innovation Center of Advanced Microstructures and School of Physics Nanjing University Nanjing 210093 P. R. China; ^2^ Institute of Atom Manufacturing Nanjing University Suzhou 215163 P. R. China; ^3^ Nanjing Institute of Atom Manufacturing Nanjing 211800 P. R. China; ^4^ College of Science National University of Defense Technology Changsha 410073 P. R. China; ^5^ Hunan Key Laboratory of Extreme Matter and Applications National University of Defense Technology Changsha 410073 P. R. China; ^6^ Key Laboratory of Optoelectronic Materials and Devices Institute of Semiconductors Chinese Academy of Sciences Beijing 100083 P. R. China; ^7^ Hunan Research Center of the Basic Discipline for Physical States National University of Defense Technology Changsha 410073 P. R. China; ^8^ National Laboratory of Solid State Microstructures and College of Engineering and Applied Sciences Nanjing University Nanjing 210093 P. R. China

**Keywords:** agglomeration resistance, cluster beam implantation, oxidation resistance, polymer packaging, universal protection

## Abstract

Size‐selected gas‐phase aggregated metal clusters have long been considered as embryos of materials. However, a significant proportion of these clusters are susceptive to damage when exiting vacuum. Consequently, effective protection is highly desirable. Here a cluster packaging and protection strategy is presented based on controllable cluster beam implantation into polymethyl methacrylate (PMMA). In this strategy, the size selection of gas‐phase clusters and the synergistic heating of PMMA during implantation are combined, thereby achieving nondestructive packaging of atomically precise cluster under soft‐landing conditions. The packaged clusters exhibit robust and universal protection. Robustness is reflected in the unaltered oxidation resistance of Mo clusters after exposure to air for over 30 days, as well as the effective agglomeration resistance at temperatures up to 100 °C or in liquid. Universality is demonstrated by the successful protection of a wide range of size‐selected clusters, including Mo clusters ranging from Mo_2057_ to Mo_6_, and Ta_2057_, Cu_923_, W_55_, (Re–Mo)_147_ clusters. This protection is attributed to both a stable, albeit weak PMMA‐Mo bonding, forming Mo─O─C─C species that stabilize the clusters, and the direct implantation of gas‐phase cluster into solid‐phase PMMA. This helps pave the way for further investigation and applications of gas‐phase metal clusters.

## Graphical abstract

Gas‐phase‐aggregated metal clusters are packaged and well‐protected in poly(methyl methacrylate) (PMMA) through controlled beam implantation. The packaged precise clusters show robust protection against oxidation and agglomeration. This protection also demonstrates universality, as it successfully protects metal clusters composed of various elements (including Mo, Ta, Cu, W, and Re) and a broad range of atom numbers (2057‐6).

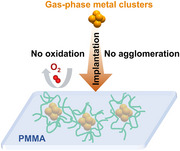

## 1 Introduction

Isolated, atomically precise metal clusters have long been regarded as embryos of new materials,^[^ ^1^ ^]^ and are widely applied in optics, catalysis, electronics, magnetics, and superconductivity.^[^ ^2^ ^]^ Importantly, the alteration of size or even an atom can lead to substantial alterations in their properties. Among them, the synthesis of atomically precise gas‐phase metal clusters via gas‐phase condensation and size selection is applicable to a broad range of material types^[^ ^3^ ^]^ and cluster sizes.^[^ ^4^ ^]^ However, these gas‐phase clusters, even some with magic numbers, can exhibit flexible and reactive intra‐clusters metallic bonds, that may induce agglomeration when the clusters are in close proximity and lack sufficient stabilizations from the external environment;^[^ ^5^ ^]^ Furthermore, for most non‐noble metal clusters, oxidation is inevitable upon exposure to air. Consequently, these gas‐phase clusters are prone to ubiquitous oxidation and agglomeration when exiting the vacuum,^[^ ^6^ ^]^ which significantly hinders the development and broader application of this general gas‐phase method.^[^ ^5c^ ^]^ Therefore, for the purpose of further investigation and potential applications, it is crucial to develop robust and universal protection strategy for these size‐selected gas‐phase metal clusters while preserving their size precision.^[^ ^7^ ^]^

Efforts to meet this demand have been ongoing for over three decades. Cryogenically solidified gases,^[^ ^8^ ^]^ ion liquids^[^ ^9^ ^]^ or ligand‐dissolved solutions^[^ ^10^ ^]^ have been sequentially employed for the stabilization of gas‐phase‐aggregated metal clusters, providing physical or chemical protection. However, these approaches all lack robust and universal protection. First, for cryogenic solidified gases, increasing the temperature above their melting points leads to inevitable cluster agglomeration and oxidation.^[^ ^8a^ ^]^ Second, for ion liquids, the limited cluster protection arises particularly in liquid environments,^[^ ^9b^ ^]^ leading to poor size distribution by agglomeration. Meanwhile, few research focuses on the oxidation resistance. Third, for ligand‐dissolved solutions, although ligands are widely used in chemosynthesis, they act as both stabilizing adsorbate and efficient etchant^[^ ^11^ ^]^ for these gas‐phase aggregated clusters, which complicates achieving universal protection and may cause additional destruction.^[^ ^1b^ ^]^ Moreover, some reports indicate that certain polymers also demonstrate potential for cluster protection, but still none have met the requirements for universal and robust cluster protection. For example, polymer‐stabilized Fe clusters through wet chemical reduction show no oxidation, but lacking universality and control on cluster size and elements;^[^ ^12^ ^]^ polymer‐stabilized size‐selected Au and Ag clusters, which are prepared via deposition onto polymers and following dissolution in a solvent, show suppressed agglomeration,^[^ ^13^ ^]^ but this study did not involve more oxidizable non‐noble metal clusters, and the introduction of the liquid phase results in a deterioration of the size distribution compared to its initial state. However, this prompts us to conjecture whether it is possible to integrate the above‐mentioned methods to take advantage of their respective strengths. Specifically, gas‐phase synthesis with size‐selection can achieve precise control over the size and composition of clusters, while certain solid‐phase polymers can provide effective protection for these clusters and avoid further agglomeration. The challenge lies in implementing this strategy effectively. Fortunately, the well‐developed method of implanting gas‐phase aggregated nanoclusters into polymers offers a promising solution.^[^ ^14^ ^]^ Under precisely controlled conditions, these implanted clusters can stably integrate into the polymer matrix to a depth of several tens of nanometers, resulting in various nanocomposites with superior properties regulated by both polymers and nanoclusters.^[^ ^14^, ^15^ ^]^ However, current researches have paid little attention on the possibility to realize effective protections for the implanted clusters, especially those size‐selected ones, particularly against oxidation and agglomeration. Moreover, an improved implantation process is required to minimize potential damage to these precise clusters.

Here, we show successful packaging of size‐selected gas‐phase metal clusters through controlled beam implantation into poly(methyl methacrylate) (PMMA), thereby achieving universal and robust protection. First, building on cluster beam implantation process, we combine size‐selected metal clusters with precisely controlled implantation under soft‐landing conditions, thereby nondestructively packaging the atomically precise clusters in PMMA. Second, our research demonstrates that clusters packaged by PMMA, particularly non‐noble metal clusters, exhibit robust protection against oxidation and agglomeration. This protection is consistently observed across various elements and cluster sizes, indicating its universality. Third, the achieved protection is attributed to stable, albeit weak PMMA‐cluster bonding and the compact surrounding environment provided by solid PMMA. Given the unique properties of size‐selected metal clusters and the extensive applications of metal nanoparticle‐polymer nanocomposites, this universal and robust protection for gas‐phase precise metal clusters, especially non‐noble metal clusters, help pave the way for their further investigations and applications.

## 2 Results and Discussion

### 2.1 Packaging Strategy of Size‐Selected Clusters into PMMA through Controllable Beam Implantation

**Figure 1:**
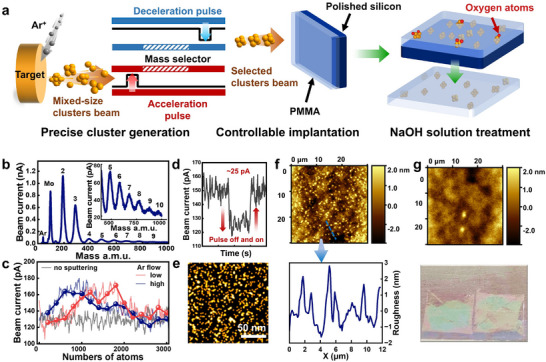
Successful packaging of clusters into PMMA. a) Schematic representation of a modularized process for cluster implantation, including precise cluster generation, controllable beam implantation, separation and NaOH solution treatment procedures. The gray spheres represent the argon gas used for magnetron sputtering, the red spheres represent the oxygen atoms, and the agglomerated orange spheres represent the generated Mo clusters. b) Mass spectrum of size‐selected small Mo clusters using a time‐of‐flight mass selector. c) Gray, red, and blue curves represent the background signal, as well as the mass spectra of Mo clusters in a large mass distribution under different Ar flow rates, respectively. The line charts with markers are constructed by selecting points in the mass spectra at intervals of every 200 atoms to provide clearer presentation for broad peaks. d) Beam current of generated Mo_923_ clusters by deactivating and then activating the mass‐selective pulse. e) FIB‐TEM images of the cross sectional morphology of Mo_923_–PMMA nanocomposite, which demonstrate the successful implantation of Mo_923_ clusters into a PMMA membrane. The packaged clusters exhibit a dispersed and uniform distribution, albeit with slight agglomeration. f) AFM image of Mo_923_‐implanted PMMA membranes before NaOH solution treatment and the acquired surface roughness along the blue dashed line. g) AFM image of Mo_923_‐implanted PMMA membranes after NaOH solution treatment. The final obtained Mo_923_–PMMA membranes are also shown at the bottom.

A modularized process comprising three sequential procedures, namely, precise gas‐phase cluster generation, controllable cluster beam implantation, and NaOH solution treatment, was developed (**Figure** **1**a) to package clusters in PMMA. First, metal clusters were precisely generated through a magnetron sputtering gas‐phase condensation cluster source equipped with a time‐of‐flight mass selector.^[^ ^4b^ ^]^ A typical mass spectrum of the generated small Mo clusters is shown in Figure 1b, demonstrating discrete cluster peaks with a typical mass resolution of m/Δm≈10. Moreover, the mass spectra of Mo clusters in a large mass distribution reveal broadened peaks (Figure 1c).^[^ ^4a^ ^]^ The range of these broad peaks represent the size ranges of synthesizable Mo clusters, which are modulated by the condensation conditions within our cluster source. Specifically, under low Ar flow rate condition (107 standard cubic centimeter per minute (sccm)), clusters experience a longer transit time in the condensation chamber, allowing them to grow larger.^[^ ^16^ ^]^ Conversely, higher Ar flow rate (195 sccm) results in smaller clusters. In this way, Mo clusters can be size‐selected varying in size from a few to thousands of atoms. For example, for Mo_923_ clusters, we have 923 ± 47, under the mass resolution of ≈10. Additionally, Figure 1d demonstrates a typical beam current of ≈25 pA for size‐selected Mo_923_ clusters by deactivating and then activating the mass‐selective pulse. Subsequently, controllable beam implantation was performed in a vacuum‐interconnected implantation system under 10^−5^ Pa, targeting PMMA that had been previously spin‐coated on a n‐doped silicon slice. To avoid cluster destruction during the implantation, clusters were vertically implanted with a soft‐landing energy lower than the binding energy per atom for clusters.^[^ ^17^ ^]^ Meanwhile, to ensure the successful cluster implantation under soft landing condition, the low‐temperature processability of PMMA was utilized through synergistic heating from both cluster beam implantation^[^ ^18^ ^]^ and external heating. Specifically, PMMA was externally heated to 50 °C for Mo clusters larger than 55 atoms, and to 70 °C for smaller clusters. Additionally, preannealing of PMMA should be avoided before implantation to maintain its viscous state, which facilitates the implantation of clusters in PMMA.^[^ ^15c^ ^]^ The successful packaging of clusters into PMMA membranes was confirmed using focused ion beam‐transmission electron microscopy (FIB‐TEM) analysis. The results revealed the cross sectional morphology of the Mo_923_–PMMA nanocomposite, demonstrating that Mo_923_ clusters were dispersed uniformly in PMMA (Figure 1e). This observation was consistent with recent findings^[^ ^15^, ^19^ ^]^ and our theoretical simulation result (Figure S1, Supporting Information). Notably, the PMMA membrane almost melted during the FIB process, resulting in the observed slight agglomeration of Mo_923_ in Figure 1e. After the implantation, 0.1 mol L^−1^ NaOH solution treatment was carried out to detach the PMMA membrane from the silicon slice and remove oxidized Mo clusters residual on the surface of PMMA. In this procedure no detectable destruction to PMMA membrane was observed (Figure S2, Supporting Information).^[^ ^20^ ^]^ The successful removal of surface‐remaining oxidized Mo clusters was confirmed via atomic force microscopy (AFM). As shown in Figure 1f, prior to NaOH solution treatment, oxidized Mo_923_ clusters were partially embedded on the PMMA surface,^[^ ^15c^ ^]^ exhibiting a relevant height of ≈2–5 nm. However, after NaOH solution treatment, almost all the oxidized Mo_923_ on the PMMA surface were completely etched away (Figure 1g). Additionally, a photograph of the actual acquired Mo cluster–PMMA membranes (10 × 10 mm^2^) is shown in Figure 1g.

In brief, we demonstrate a successful packaging of size‐selected cluster in PMMA building on the beam implantation process. This is mainly attributed to the significant surface energy differences between metal clusters and PMMA,^[^ ^14b^ ^]^ as well as the synergistic heating on PMMA via both cluster beam implantation^[^ ^18^ ^]^ and external heating based on the low‐temperature processability of PMMA. Notably, this combined heating approach allows for the implantation of size‐selected clusters under controllable soft‐landing conditions to keep the selected cluster size. The extra NaOH solution treatment also removes any clusters that failed to implant in PMMA successfully.

### 2.2 Nondestructively Packaged Mo Clusters in PMMA with Universal and Robust Oxidation Resistance

**Figure 2:**
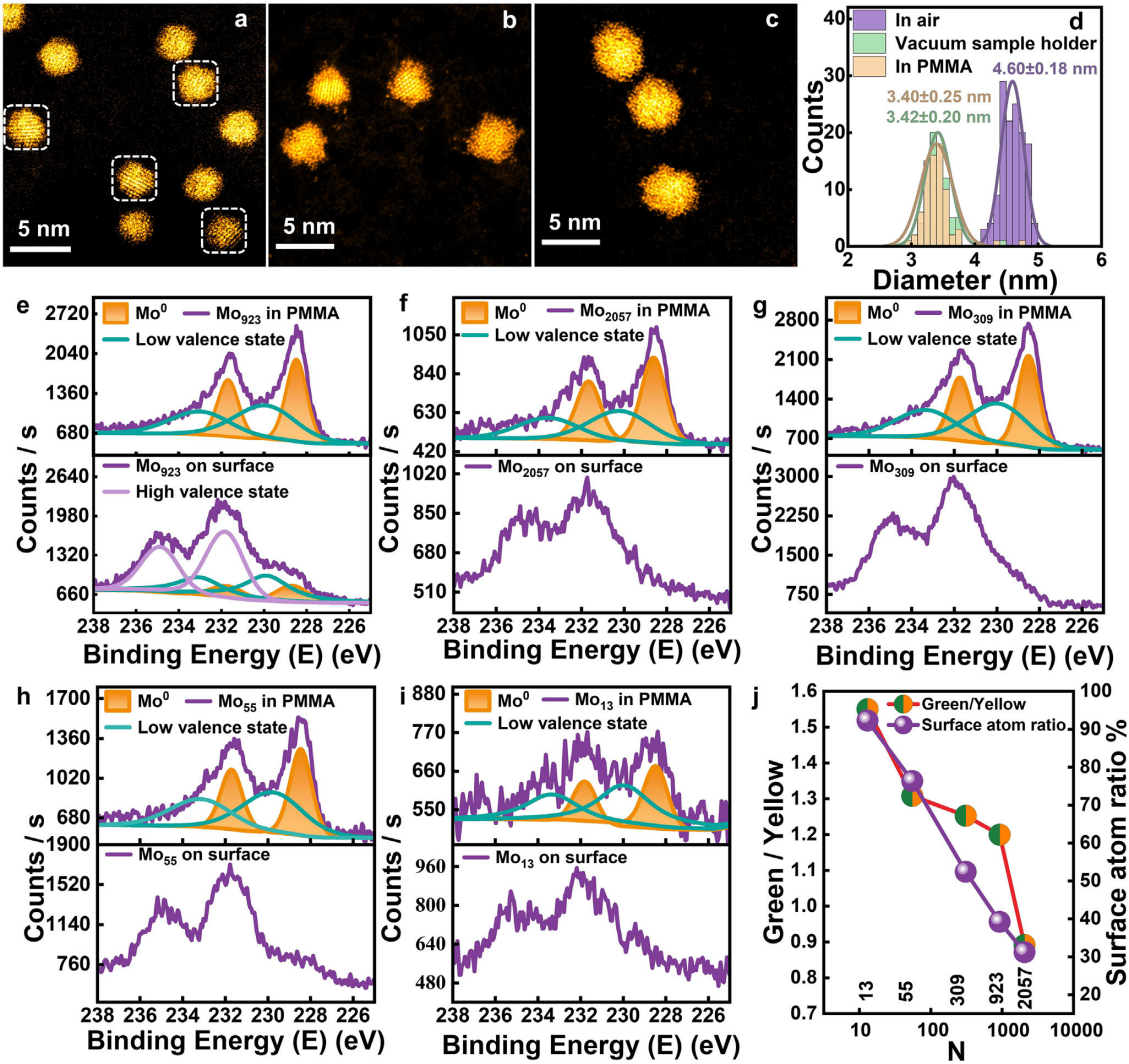
Evidence of robust oxidation‐resistant Mo clusters packaged in PMMA with alternative number of atoms. a–c) STEM images of Mo_923_ clusters packaged in PMMA, protected in a vacuum‐transfer sample holder and directly exposed to the atmosphere. Packaged Mo_923_ clusters with ordered structures are highlighted within white dashed frames. d) Size statistics of the Mo_923_ clusters obtained under the aforementioned three preparation conditions. e) Mo 3d XPS spectra acquired from Mo_923_ clusters on the surface of (bottom) and within the PMMA (top). Three distinct pairs of peaks, colored light‐violet, green, and yellow, correspond to the high valence state resulting from oxidation, the low‐ and the zero‐valence states of the Mo XPS signal, respectively. f–i) Mo 3d XPS spectra of packaged Mo clusters with various atom numbers (2057, 309, 55, and 13) obtained from the surface of and within the PMMA. All the packaged clusters within the PMMA are protected against oxidation. j) The ratio of green to yellow peak area, as well as the surface atom ratio of Mo clusters, as functions of number of atoms of Mo clusters.

In spherical aberration‐corrected scanning transmission electron microscopy (STEM), PMMA‐packaged size‐selected Mo_923_ clusters exhibit compact and ordered structures (white dashed frame) with a uniform size distribution (**Figure** **2**a), suggesting the nondestructive packaging of Mo_923_ clusters under soft‐landing implantation condition. In comparison, Mo_923_ clusters maintained in the vacuum‐transfer sample holder, which have not been exposed to air, also exhibit similar ordered and uniform morphology (Figure 2b); while Mo_923_ clusters exposed in the atmosphere undergo serious oxidation, exhibiting an amorphous morphology with a significantly larger size (Figure 2c). The size statistics in Figure 2d clearly indicate that the diameter and size distribution of the PMMA‐packaged and vacuum‐transfer sample holder‐protected Mo_923_ clusters are nearly identical (3.40 ± 0.25 and 3.42 ± 0.20 nm, respectively), and are much smaller to those of the Mo_923_ clusters oxidized in the atmosphere (4.60 ± 0.18 nm).

Furthermore, X‐ray photoelectron spectroscopy (XPS) was conducted to investigate the valence state of the PMMA‐packaged Mo clusters. Notably, to comprehensively investigate the evolution of the valence states, no NaOH solution treatment was carried out after implantation, resulting in the presence of oxidized Mo clusters on the PMMA surface. In the bottom image of Figure 2e, the Mo 3d XPS spectra of Mo_923_ from the PMMA surface shows complex peaks with a shoulder located at a low binding energy, and three pairs of Mo 3d peaks can be resolved. The main pair of peaks, colored light violet, indicates the oxidation of Mo_923_ clusters on the PMMA surface, with 3d_5/2_ peaks located at ≈232 eV.^[^ ^21^ ^]^ Additionally, a pair of yellow‐filled peaks exhibit a binding energy of ≈228.6 eV for its 3d_5/2_ peak, indicating the presence of Mo ingredients with zero valence.^[^ ^22^ ^]^ The third pair of peaks, colored green, is situated between the light violet and yellow peaks, with 3d_5/2_ peaks located at 229.9 eV, suggesting that some Mo ingredients are in a lower valence state distinct from oxidation.^[^ ^23^ ^]^ Through the in situ Ar^+^ etching process, the surface layer of PMMA is removed to expose initially packaged Mo_923_ clusters, and the top image of Figure 2e illustrates a prominent alteration in the Mo XPS spectra. First, the complete disappearance of the hypervalent Mo component at 232 eV indicates the absence of oxidized Mo_923_. Second, the remaining peaks can also be resolved as two pairs of peaks located within the same energy range as those in the bottom image of Figure 2e. These peaks are also colored yellow and green, representing zero‐ and low‐valence‐state Mo constituents, respectively. We further excluded the possibility that Ar^+^ etching is responsible for both the disappearance of hypervalent Mo and the increased proportion of zero valence‐state Mo^[^ ^24^ ^]^ in Figure S3 (Supporting Information). Therefore, through XPS and STEM results, it is convinced that PMMA packaging through controlled implantation effectively suppresses the oxidation of Mo_923_ clusters. In addition to Mo_923_, the XPS spectra of size‐selected Mo clusters ranging from 2057 to 13 atoms are depicted in Figure 2f–i. These spectra exhibit a consistent trend of change analogous to that observed for Mo_923_ both before and after Ar^+^ etching. Therefore, it is evident that for PMMA‐packaged Mo clusters containing alternative number of atoms, the hypervalent Mo XPS peaks induced by oxidation all vanish, whereas the yellow‐ and green‐colored XPS peaks recur. Furthermore, long‐term oxidation resistance was demonstrated, as no significant changes in XPS were observed after exposing PMMA‐packaged Mo_923_ to atmospheric conditions for over a month (Figure S4, Supporting Information). Thus, the PMMA‐packaged Mo clusters exhibit robust oxidation resistance over a broad range of atom numbers.

As for the repeatable lower valence state of the Mo ingredients (green‐colored peaks), the possibilities that it may arise from partial and slow oxidation of Mo by oxygen, or partial reduction of hypervalent Mo oxides through Ar^+^ etching^[^ ^24^ ^]^ are also excluded (Figures S5 and S6, Supporting Information). However, the synchronized trend observed between the ratio of the green peak area to the yellow peak area and the surface atom ratio^[^ ^25^ ^]^ of clusters versus the number of atoms (Figure 2j) indicates its possible connections to the surface atoms of Mo clusters.

Notably, considering that the mean free path of photoelectrons is ≈5 nm in polymers,^[^ ^26^ ^]^ the shoulder of surface Mo 3d XPS peaks, namely the yellow and green pairs of peaks (observed in Figure 2e–i), is likely attributable to unoxidized Mo clusters that are shallowly but completely packaged in PMMA. This assertion is further supported by Figure S7 (Supporting Information), which demonstrates that after NaOH solution treatment to remove oxidized Mo clusters residual on PMMA surface, the hypervalent Mo XPS peaks resulting from oxidation are absent from the surface Mo 3d XPS spectra, whereas the green and yellow pairs of peaks remain clearly detectable.

In summary, STEM and XPS results provide compelling evidence that size‐selected Mo clusters are nondestructively packaged and protected against oxidation in PMMA under soft‐landing implantation. This protection is universal for Mo clusters ranging from 2057 to 13 atoms, and provides robust oxidation resistance for over a month. Notably, this remarkable oxidation resistance even works for shallowly packaged Mo clusters. Furthermore, the low‐valence‐state Mo green XPS peaks is probably associated with surface atoms of Mo clusters.

### 2.3 Interaction Between Mo Clusters and PMMA with Enhanced Agglomeration Resistance

**Figure 3:**
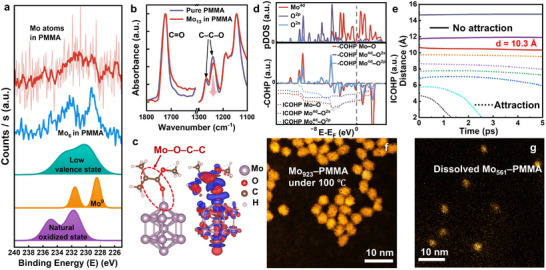
PMMA and Mo clusters bonding with enhanced agglomeration resistance. a) From the bottom up, the graphs are naturally oxidized high‐valence‐state Mo 3d XPS spectra fitted from XPS spectra of Mo_309_ on the PMMA surface, with the background subtracted; zero‐ and low‐valence‐state Mo 3d XPS spectra fitted from XPS spectra of Mo_309_ in PMMA, with the background subtracted; Mo 3d XPS spectra of Mo_6_ and low‐concentration Mo atoms in PMMA, respectively. Additionally, a red‐colored smoothing line acquired from the XPS spectra of the Mo atoms in PMMA is also present because of the poor signal‐to‐noise ratio. b) FTIR spectra of the Mo_13_–PMMA nanocomposite and pure PMMA normalized at 1724 and 1143 cm^−1^, respectively. c) The most stable configuration of the adsorbed Mo_13_–MMA complex and the charge transfer between Mo_13_ and MMA are depicted, with blue regions representing electron depletion and red regions representing electron accumulation. d) Projected density of states (pDOS), crystal orbital Hamilton population (COHP) and integrated COHP (ICOHP) analyses of the Mo_13_–MMA complex, with the Fermi level set at 0 eV in the dashed line. e) The dynamics of two Mo_13_ clusters initiated with varying nearest cluster distances. The solid lines represent unattractive distances (greater than 10.3 Å), whereas the dotted lines indicate attractive distances. f) STEM image of the Mo_923_–PMMA nanocomposite subjected to a temperature of 100 °C. g) STEM image of Mo_561_ clusters redissolved from the Mo_561_–PMMA nanocomposite by anisole.

We further investigate the interaction between PMMA and the packaged Mo clusters in this section. The Mo 3d XPS spectra of the PMMA‐packaged Mo_6_ clusters and low‐concentration Mo atoms are presented in **Figure** **3**a. In the case of packaged low‐concentration Mo atoms in PMMA, the XPS spectra exhibit a lower binding energy than the fitted violet‐colored curve which represents the native oxidized state of Mo in Mo_309_, but show good agreement with the fitted green‐colored curve which represents a lower valence state of Mo in Mo_309_. Given that low‐concentration Mo atoms packaged in PMMA can only interact with PMMA or maintain zero‐valent state, we hypothesize that the green‐colored XPS peaks arise from interactions between Mo atoms and PMMA at the cluster surface. This hypothesis is further supported by the synchronized increase in both the proportion of the green XPS peak area and the surface atom percentage of Mo clusters, as shown in Figure 2j. Moreover, an analogous phenomenon was described when low concentrations of Al atoms are implanted in polyethyleneterephthalate (PET).^[^ ^26^ ^]^ In that case only the high Al valence state related to the Al─O─C species was observed. Similarly, in the case of packaged ultrasmall Mo_6_ without a core,^[^ ^25^ ^]^ no oxidation to hypervalent state occurs. However, its binding energy exceeds that of the fitted zero‐valence state of Mo in Mo_309_ (yellow curve) and other larger Mo clusters (Figures S8 and S9, Supporting Information), but is closer to that of Mo atoms. Therefore, ultrasmall clusters packaged in PMMA can also be protected against oxidation, but their overall valence state may be influenced by interactions with PMMA. In addition to XPS analysis, Fourier transform infrared (FTIR) spectroscopy is utilized to detect potential alterations in the vibration patterns of the PMMA upon Mo cluster packaging (Figure 3b). The packaging of Mo_13_ clusters in PMMA induces changes in the relative intensities of the infrared bands at 1241 and 1271 cm^−1^, which correspond to the cooperative symmetric and antisymmetric stretches of the C─C─O group in PMMA, respectively.^[^ ^27^ ^]^ Furthermore, the absence of absorption bands at 1702 and 1674 cm^−1^ indicates that no detectable hydrolysis of C = O into COOH and COO^−^ occurred.^[^ ^27^ ^]^ These observations indicate the conformational alterations in the polymer chain to form Mo─O─C─C species on the surface of the Mo clusters.^[^ ^27a^ ^]^

Moreover, theoretical calculations are employed to investigate the interactions between the PMMA and Mo clusters. It is calculated that the methyl methacrylate (MMA) molecule adsorbs onto the top site of the Mo_13_ cluster upon relaxation, forming a Mo─O─C─C bond (Figure 3c), which aligns with the aforementioned FTIR result. Furthermore, charge difference analysis and both the Mulliken and Löwdin charge population results (Figure 3c and **Table** **1**) reveal electron transfer from the Mo cluster to the MMA molecule, resulting in a slight increase in the valence state of the Mo atoms, which is consistent with the green‐colored XPS peaks. We further conduct an in‐depth analysis of the characteristics of this bonding. A comparison of the projected density of states (pDOS) and the crystal orbital Hamilton population (COHP) below the Fermi surface demonstrated that the Mo─O─C─C bond mainly consists of s–d and p–d electron pairs (Figure 3d). The COHP analysis demonstrates that these electron pairs exhibit a mixed character of bonding and antibonding states upon energy, but they exhibit a bonding state with a negative value of the integrated crystal orbital Hamiltonian population (ICOHP), indicating an albeit weak chemical bonding (Table S1, Supporting Information). Similar results regarding the PMMA–Mo_6_ interaction are also presented in Figures S10–S12 (Supporting Information) and Table S2 (Supporting Information). Hence, based on the repeatedly appeared green‐colored XPS peaks for various Mo clusters, and the results from theoretical calculations, we conclude that a stable, albeit weak PMMA‐Mo cluster bonding are generally present.

**Table 1:** Mulliken and Löwdin charge analysis (MCA and LCA) of the Mo_13_–MMA complex. In both the MCA and LCA, positive values indicate electron depletion, whereas negative values indicate electron accumulation.

Molecule	MCA (e)	LCA (e)
Mo_13_	0.45	0.39
MMA	−0.45	−0.39

Notably, the bonding between the Mo clusters and PMMA is likely to increase the agglomeration resistance of the clusters because of the steric hindrance effect. In Figure 3e, molecular dynamics (MD) simulations reveal that the nearest interaction distance between two Mo_13_ clusters is ≈10.3 Å in vacuum. However, this distance can be easily exceeded through bonding to MMA, not to mention the rigid solid PMMA chains in real situations. Additionally, similar enhanced aggregation resistance is achieved for the Mo_6_ clusters (Figure S13, Supporting Information). Experimentally, this enhanced agglomeration resistance of Mo clusters is further demonstrated. In Figure 3f, the Mo_923_ clusters packaged in PMMA at 100 °C just below the glass temperature (T_g_) of PMMA^[^ ^28^ ^]^ exhibit limited agglomeration. In contrast, Mo_923_ clusters directly deposited on carbon films at the same temperature significantly agglomerated into larger nonspherical clusters (Figure S14, Supporting Information). Furthermore, the Mo_561_–PMMA nanocomposite dissolved in anisole (Figure S15, Supporting Information) exhibited only minor agglomeration (Figure 3g), despite experiencing strong attractive forces among the clusters in the liquid suspension. Therefore, this PMMA‐Mo cluster bonding in solid PMMA matrix suppresses the cluster interactions and enhances agglomeration resistance.

Therefore, it is concluded that PMMA forms stable, albeit weak, Mo─O─C─C chemical bonds with packaged Mo clusters and elevates the valence state of some Mo atoms. Additionally, this bonding in solid PMMA matrix enables enhanced agglomeration resistance of clusters even in quite extreme environments.

### 2.4 A Universal Strategy for Clusters Protection and the Preserved Clusters Properties

**Figure 4:**
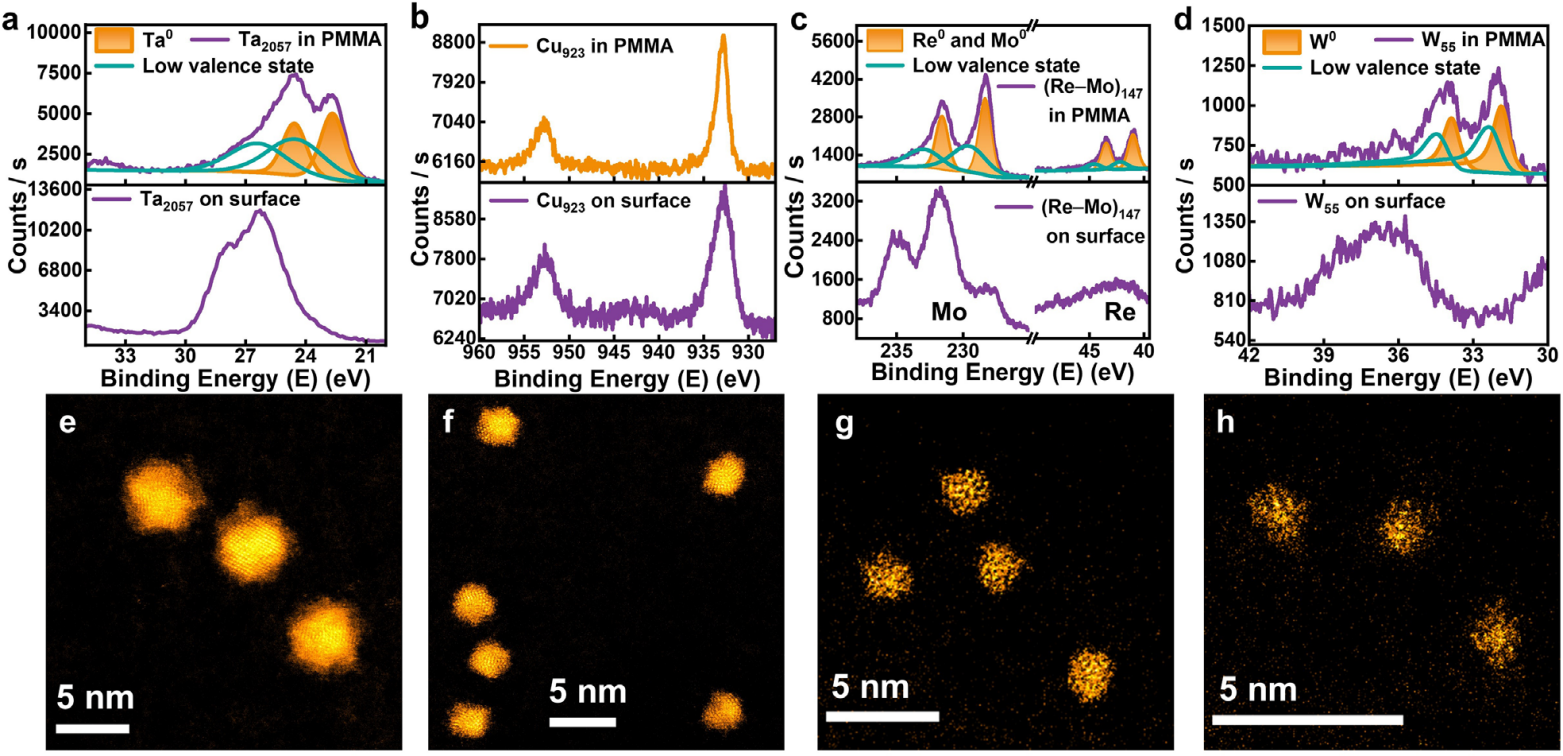
A universal protection for PMMA‐packaged metal clusters. a–d) XPS spectra of Ta_2057_, Cu_923_, (Re–Mo)_147_ and W_55_ clusters on the surface of and within the PMMA. Through PMMA packaging, the XPS signals associated with hypervalent oxidized metals all disappear, whereas the XPS signals corresponding to zero‐ and lower‐valence‐state peaks remain. e–h) STEM images of the PMMA‐packaged clusters mentioned above.

The universality of this protection strategy except for Mo clusters is demonstrated by investigating PMMA‐packaged clusters containing different elements and number of atoms. To obtain clear STEM images, clusters larger than 55 atoms are selected for improved contrast. In **Figure** **4**a–d, the XPS results indicate that clusters of other elements (Ta_2057_, Cu_923_, W_55_) and even alloys ((Re–Mo) _147_) can also be protected in PMMA without oxidation. Notably, (Re–Mo)_147_ refers to size‐selected alloyed Re–Mo clusters with a central atomic mass 147 times the average atomic mass of Re and Mo. To be more specific, for Ta_2057_, (Re–Mo)_147_ and W_55_ clusters, the absence of high valence states in the XPS spectra of the PMMA‐packaged Ta_2057_, (Re–Mo)_147_, and W_55_ clusters signifies their increased oxidation resistance. The remaining XPS peaks can also be resolved into two pairs of peaks corresponding to the unoxidized and low‐valence state ingredient in clusters;^[^ ^24^, ^29^ ^]^ these observations are analogous to the phenomenon observed in packaged Mo clusters. Furthermore, for Cu_923_, the disappearance of the satellite peak located at ≈943 eV and a reduced FWHM of the Cu 2p_3/2_ peak clearly indicate the oxidation resistance of the PMMA‐packaged Cu_923_.^[^ ^30^ ^]^ The corresponding STEM images are also presented in Figure 4e–h, revealing the absence of oxidation and agglomeration of the PMMA‐packaged clusters. The aforementioned four clusters all show homogeneous size distributions without agglomeration, with sizes notably smaller than those exposed in air with oxidation (Figure S16, Supporting Information). Additionally, for larger clusters, namely, Ta_2057_ and Cu_923_, ordered structures are easy to observe. Thus, a universal packaging and protection strategy is proposed for clusters comprising varying numbers of atoms and elements. Considering the potential applications of packaged clusters as well as cluster‐polymer nanocomposites, we present two simple examples in Figures S17 and S18 (Supporting Information) to show the preserved optical properties. First, the Cu_923_–PMMA membrane exhibited stable optical absorption over a month without UV–O_3_ treatment,^[^ ^31^ ^]^ which was recently used to create dense oxide layers to prevent further oxidation of the Cu clusters. Second, the Mo_6_–PMMA membrane displays sharp emission at ≈550–650 nm under 350 nm excitation compared with the photoluminescence spectra of the pure PMMA membranes.

### 2.5 Discussion and Experimental Prospect

Building on the well‐established and widely applicated cluster beam synthesis of polymer composites with nanoparticles, our proposed PMMA packaging strategy offers universal and robust protection for size‐selected clusters. This strategy possesses several key characteristics. First, in terms of precise cluster packaging, to prevent potential damage of clusters, external heating of PMMA is carried out during cluster beam implantation. Consequently, the flexibility of polymer chains is further increased and the clusters can penetrate deeper than at room temperature (RT) under the same implantation energy.^[^ ^15^, ^32^ ^]^ Therefore, the implantation of clusters under soft landing conditions is further guaranteed. Additionally, clusters are size‐selected in the gas phase before nondestructively packaged into solid PMMA, thereby achieving atomically precise cluster size control; Second, regarding the achieved cluster protection against oxidation and agglomeration, solid PMMA not only serves as a compact and rigid structural framework surrounding clusters to avoid liquid environment and suppress the cluster interactions, but also establishes stable, albeit weak bonding with the packaged clusters. Specifically, the bonding effect of PMMA on clusters resembles one of the key functions of chemical ligands: acting as a stable adsorbate to stabilize the clusters. However, given that the clusters are size‐selected and directly packaged into solid‐phase PMMA without destruction, our protection strategy eliminates the necessity of using strong ligands as efficient etchants for cluster size control. Consequently, the polymer PMMA alone suffices to achieve our objectives. Third, our study finally demonstrates that PMMA packaged gas‐phase metal clusters, especially those composed of non‐noble metals and precisely selected sizes, can be robustly protected against oxidation and agglomeration. Given the unique properties of gas‐phase clusters demonstrated in a large number of previous studies, as well as the widely applied nanocluster‐polymer nanocomposites with advanced optical and electrical properties prepared via beam implantation methods,^[^ ^14^, ^33^ ^]^ we hope this study offer a viable strategy for investigating the unique properties of gas‐phase metal clusters, and exploring potential applications derived from these properties.

## 3 Conclusion

We presented a detailed study on the effective protection of size‐selected gas‐phase metal clusters, which is a longstanding challenge in the field of gas‐phase cluster research. First, size‐selected clusters and synergistic heating of PMMA are integrated into the beam implantation process, ensuring nondestructively packaging of atomically precise clusters within solid PMMA under soft‐landing condition. Second, the packaged clusters exhibit both universal and robust protection. Specifically, this universal protection works for various size‐selected clusters including Mo, Ta, W, Cu or Re, with atom numbers ranging from 2057 to 6. Meanwhile, the robust protection is exemplified by the long‐time oxidation resistance of Mo_923_ more than 30 days, as well as the effective agglomeration resistance of Mo clusters at 100 °C or in a liquid environment. Third, the protection is attributed to not only the rigid environment of solid PMMA but also the stable, albeit weak PMMA‐cluster bonding as a protection layer to stabilize clusters. Considering the preservation of cluster properties such as optics, and the fantastic properties of polymer nanocomposites with nanoparticles synthesized via cluster beam process, our work is hopeful to facilitate advancements in the investigations and applications of clusters and cluster‐based nanocomposite materials, particularly those involving non‐noble metal clusters containing adjustable number of atoms.

## 4 Experimental Section

### Materials

The targets used for magnetron sputtering in the cluster source were purchased from Hebei Qinbang New Material Technology Co., Ltd., with 99.99% purity. PMMA powder (CAS: 9011‐14‐7, Aladdin) was obtained from Nanjing Shoude Biological Technology Co., Ltd. Single‐sided polished N‐doped Si substrates sliced into 10 × 10 mm^2^ pieces were purchased from Shenzhen Shunsheng Electronic Science and Technology Company. Ultrathin carbon films and GIG holey TEM grids (GIG‐1010‐3C) were purchased from Beijing Zhongjingkeyi Technology Co., Ltd.

### Preparation of the PMMA Membranes

The PMMA anisole solution was prepared by adding 5 g of PMMA powder to 100 ml of anisole. After 12 h of shaking at 70 °C, the PMMA was completely dissolved in anisole. PMMA membranes were prepared via a spin coating procedure from a 5% solution onto single‐sided polished N‐doped Si substrates. The typical spin coating parameters are 800 rpm for 10 s followed by 3000 rpm for 40 s. After spin coating, the PMMA membranes on silicon slices were dried in atmosphere for 12 h to evaporate the solvents. Notably, no annealing process was needed to maintain the viscous state of the PMMA.

### Preparation of the PMMA–Mo Clusters and Atom Nanocomposites

The size‐selected nanoclusters were produced via a magnetron sputtering gas phase condensation cluster beam source. A time‐of‐flight mass filter was used to select clusters of specific atom numbers, offering a mass resolution of M/ΔM ≈10. The size‐selected metal clusters were focused into the implantation chamber under high vacuum conditions (10^−5^ Pa) and were implanted into N‐doped silicon‐supported PMMA membranes with adjustable deposition energy to ensure soft landing conditions. TEM grids coated with ultrathin carbon films were also used to simultaneously collect directly deposited clusters.

When the clusters were implanted into the PMMA membranes, a custom heating and temperature control device was used to control the temperature, with a temperature precision of ±2%. For large clusters containing more than 55 atoms, the heating temperature is 50 °C, whereas for smaller clusters, the heating temperature is 70 °C. Since the cluster beam current was typically several to tens of pA, the total implantation process typically lasted more than 20 h.

For Mo atoms, no soft landing should be considered, and an implantation voltage of 1500 V was used for implantation into PMMA at 70 °C. The typical cluster beam current of the Mo atoms was ≈5 nA, and the deposition time lasted for 30 min.

All cluster‐PMMA samples were removed from the cluster beam source after cooling to RT.

### XPS Data Acquisition

The XPS spectra of PMMA–cluster nanocomposite were attained via X‐ray photoelectron spectroscopy (ESCALAB 250XI, Thermo Fisher). In‐depth analysis of the valence states in the sample was achieved via an etching process with the following parameters: the energy of the argon ion gun was 2000 V with a working current of 0.3 µA, the etching area was 600 × 600 µm^2^, and the etching rate was 1.0 nm s^−1^ based on Ta_2_O_5_. The etching lasted 10 s each time.

### STEM Sample Preparation and Characterization

For packaged clusters, the PMMA–cluster membranes were acquired by etching the Si substrate with 0.1 mol L^−1^ NaOH solution at RT. The floating PMMA–cluster membranes were further washed with distilled water and shredded with tweezers into fragments. A GIG holey TEM grid was used to scoop the PMMA–cluster membrane fragments, which were then dried at RT in the atmosphere.

For clusters exposed to the atmosphere, they were prepared by simultaneously depositing clusters on carbon films during the cluster implantation process. After deposition, these samples were removed directly.

For clusters in the vacuum‐transfer sample holder, a glovebox–implantation chamber‐connected system was used. After clusters were simultaneously deposited on the carbon films during the cluster implantation process, the implantation chamber was filled with N_2_, and these samples were transferred to a glovebox. After that, a vacuum‐transfer sample holder in the glovebox was used to preserve these samples.

The morphology and structure of the deposited clusters were determined with a transmission electron microscope (FEI Themis Z, Thermo Fisher) equipped with a spherical aberration probe corrector at 200 kV. A high‐angle annular dark‐field (HAADF) detector was used for imaging in STEM mode with a spatial resolution of ≈60 pm.

### FTIR Sample Preparation and Characterization

The spectroscopic performance of the PMMA membrane was characterized by a Fourier transform infrared spectrometer (IRAffinity‐1S, SHIMADZU). FTIR spectra were acquired in the wavenumber range from 600 to 4000 cm^−1^ in ATR mode, with a resolution of 2 cm^−1^.

### AFM Data Acquisition

AFM imaging was performed by Asylum Research Cypher S. Tapping‐mode AFM measurements were performed over an area of 30 × 30 µm^2^ on the sample.

### FIB Process

Cross sectional TEM samples were prepared with an FEI Helios G4 X dual‐beam system with Ga^+^ ions and further milled using Nanomill with Ar^+^ ions to prevent surface damage to the samples.

### First‐Principles Calculations

Density functional theory (DFT) calculations were performed via the Quantum‐ESPRESSO package^[^ ^34^ ^]^ with the Perdew–Burke–Ernzerhof (PBE) exchange‐correlation functional.^[^ ^35^ ^]^ Optimized norm‐conserving Vanderbilt (ONCV) pseudopotentials^[^ ^36^ ^]^ were employed, and a plane‐wave energy cutoff of 100 Ry was applied for the wavefunctions. The DFT‐D3^[^ ^37^ ^]^ van der Waals correction was incorporated to account for dispersion interactions in the adsorption systems. A vacuum spacing of at least 25 Å was used to prevent interactions between nanoclusters. Crystal orbital Hamilton population (COHP) analysis and Mulliken charge population calculations were conducted via the LOBSTER code.^[^ ^38^ ^]^

### Statistical Analysis

The size statistics of Mo_923_ clusters in PMMA, in vacuum‐transfer sample holder, and in air were carried out by measuring the size of these clusters. The numbers of clusters for size statistics were 75, 75, and 134, respectively. The fitted curve followed a normal distribution.

## Conflict of Interest

The authors declare no conflict of interest.

## Supporting information



Supporting Information

## Data Availability

The data that support the findings of this study are available from the corresponding author upon reasonable request.
