# Supplementary material for: Packaging Metal Atomic Clusters for Universal and Robust Protection through Cluster Beam Process
Source: Adv Sci (Weinh). 2025 Apr 3;12(26):2503347. doi: 10.1002/advs.202503347 (PMC12245116; doi:10.1002/advs.202503347)
Supplement: Supplementary file 1 — Supporting Information [file ADVS-12-2503347-s001.docx]

Supporting Information

**Packaging Metal Atomic Clusters for Universal and Robust Protection through Cluster Beam Process**

*Siqi Lu^[a],[b],[c]†^, Zixiang Zhao^[a],[b]^**^,[c]†^,* *Jinsen Han^[d],[e],[f],[g]†^, Jun Wang^[a]^, Yongxin Zhang^[a],[b],[c]^, Yu Du^[a],[b],[c]^,* *Fangyu Guo^[d],[e],[g]^, Zhichao Wang^[h]^, Shenghui Wang^[a],[b],[c]^, Sichen Tang^[a],[b],[c]^, Kuojuei Hu^[a],[b],[c]^, Jianguo Wan^[a]^, Jiayu Dai** *^[d],[e],[g]^, Fengqi Song** *^[a],[b],[c]^*

Supporting Figures


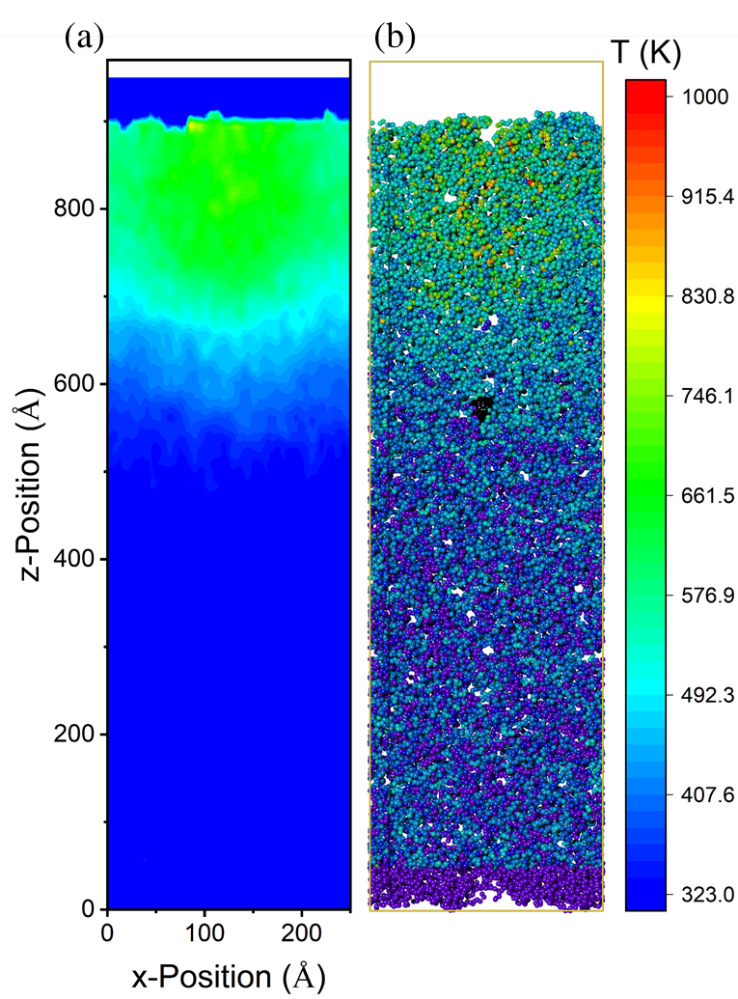


Figure S1: Implantation of gold nanocluster composed of 309 atoms (blacked circle) with kinetic energy of 100 eV to PMMA at 323 K through LAMMPS simulation. (a) The distribution of temperature after implantation. (b) Tens of implantation depth can be achieved in our simulation. The choice of gold is to avoid any possible reaction between PMMA and clusters^1^ to purely focus on the implantation process.


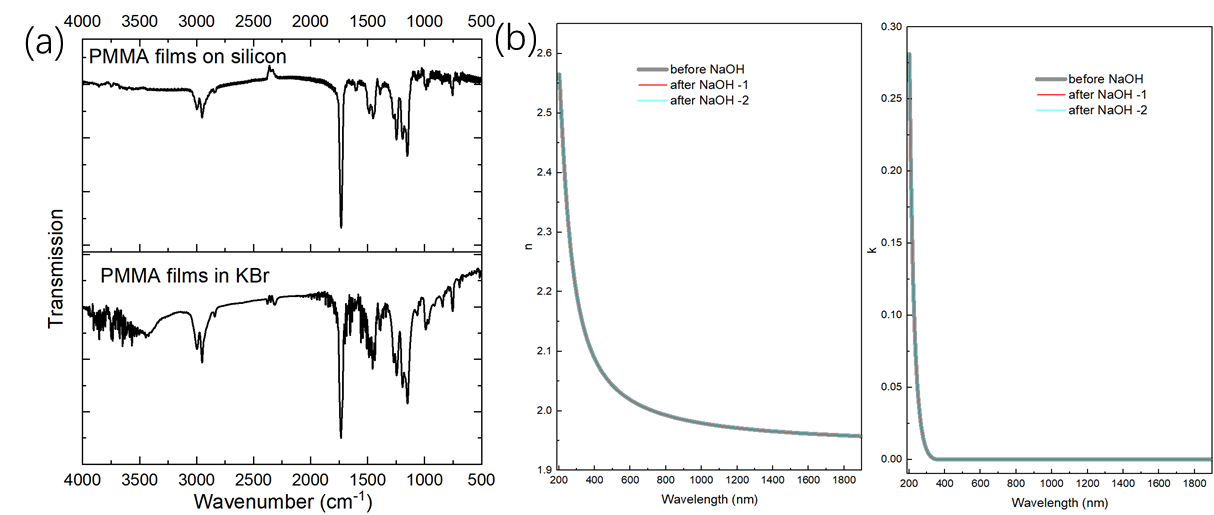


Figure S2: The etching effect of 0.1 mol/L NaOH on PMMA at room temperature. (a) FTIR result of two PMMA samples. the top image show FTIR spectra of PMMA films on double-polished intrinsic silicon by standard spin-coating method. As for the bottom one, PMMA film is peeled out from double-polished intrinsic silicon by 0.1 mol/L NaOH etching followed by tableting process with KBr powder. FTIR results show that NaOH solution have little influence on PMMA film^2^. (b) Refractive index n and extinction coefficient k of PMMA are acquired by an ellipsometer. one region is acquired before 0.1 mol/L NaOH etching and the same point as well as a neighbor region (named NaOH-1 and NaOH-2) are acquired after 1 h etching. They show almost no changes at all. Besides, the film thickness before and after NaOH etching is also calculated. before NaOH etching, the film thickness is 43.330 nm while after NaOH etching, two neighbor region show the film thickness 43.943 and 43.906 nm.


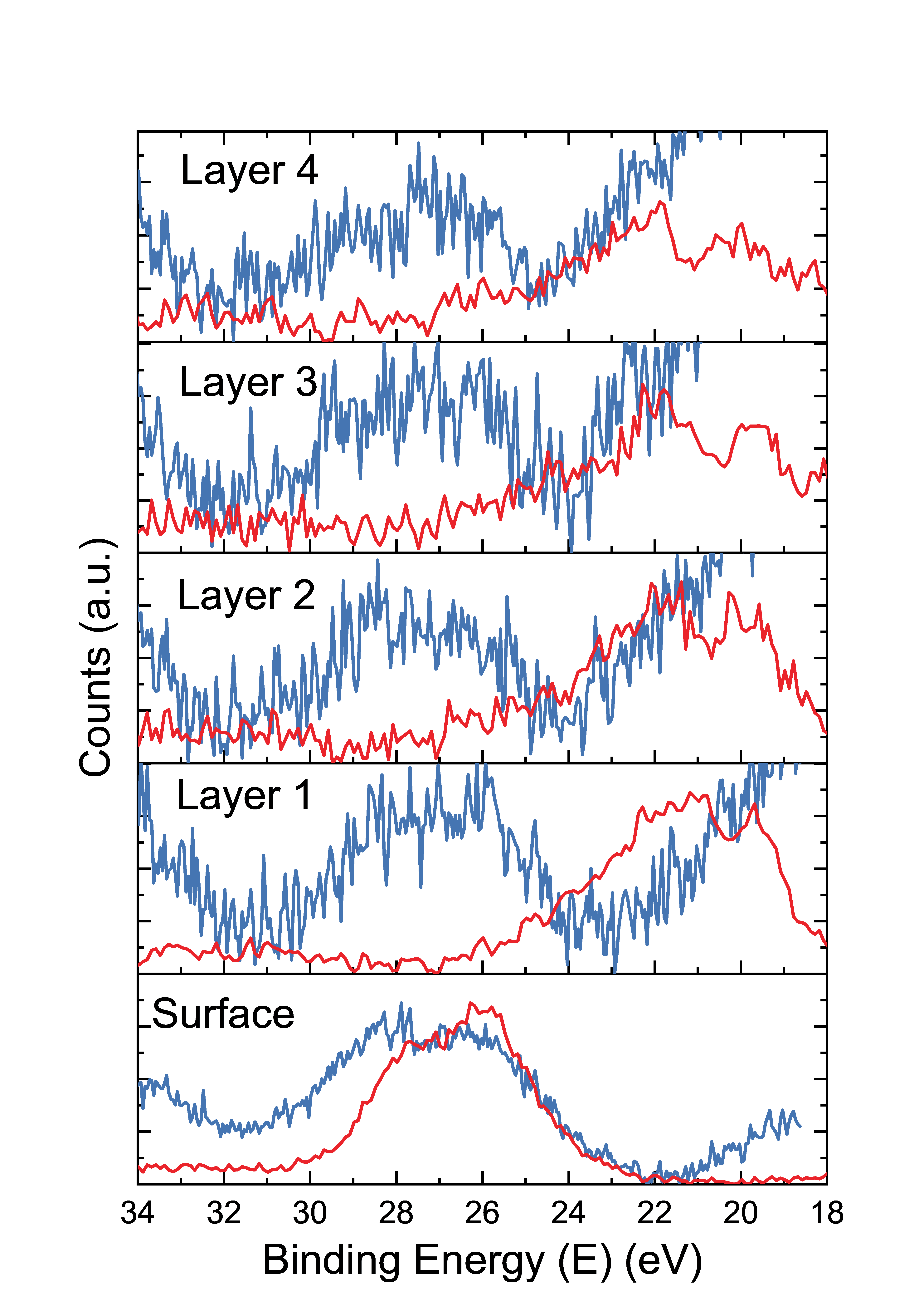


Figure S3: Ta 4f XPS spectra of pre-oxidized Ta_561_ clusters in PMMA (colored blue) as well as normally packaged Ta_561_ clusters in PMMA (colored red). At the PMMA surface, two samples show almost the same oxidation state of Ta clusters in XPS spectra. However, two XPS spectra separate with each other just in the interior of PMMA. Normally packaged Ta_561_ clusters show non-oxidized state while the pre-oxidated Ta_561_ clusters packaged in PMMA keep the oxidation state at all layers. Namely, Ar^+^ etching does no effect on the reduction.

The choice of Ta rather than Mo is due to the lower oxidation potential of Ta which is much easier than Mo to be oxidized^3^, and thus the pre-oxidization experiment can be successfully carried out in our cluster source. In pre-oxidization experiment, extra oxygen is induced into the condensation chamber of our home-made cluster source to pre-oxidated Ta clusters before packaged them into PMMA^4^.


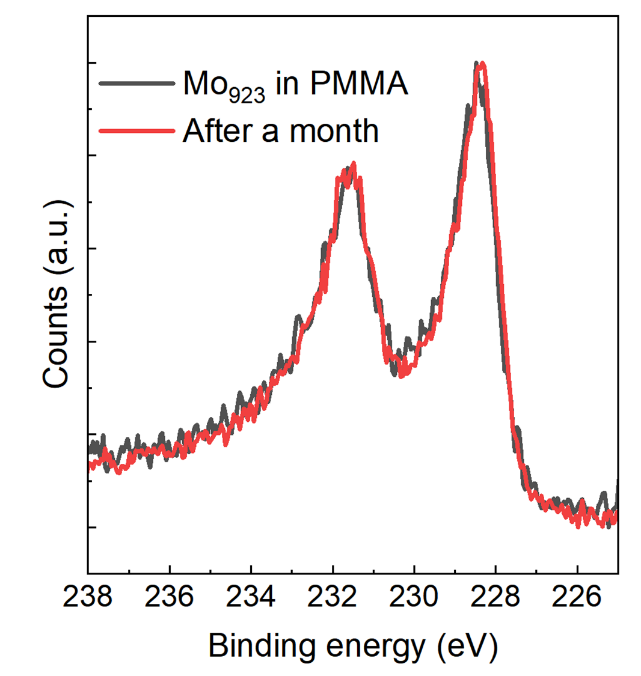


Figure S4: Normalized XPS spectra of Mo_923_-PMMA nanocomposites after a month (red line).

In Figure. S5 and S6 some experiments are carried out to investigate the possible origin of the repeatable lower valence state of Mo ingredients, i.e., the green-colored peaks. Several hypothesis are raised. Firstly, it may come from partly and slowly oxidized Mo by oxygen; Secondly, Ar^+^ etching may also cause partial reduction of Mo oxides to lower valence state^5^. Thirdly, the interaction between Mo_923_ and functional group of PMMA^6, 7^ can also increase valence state of partial Mo atoms. Since the location and proportion of green peaks keep almost unchanged even after a month and the binding energy is obviously lower than that of naturally oxidized ones (Figure S4), the first hypothesis is excluded. Additionally, the steady and repetitive appearance of green-colored pair of peaks regardless of Ar^+^ etching times (Figure S5) indicates that its origin has nothing to do with accompanying reduction effect by Ar^+^ etching. This makes the second hypothesis invalid and the green-colored peaks is most likely to originate from interaction between Mo atoms and PMMA functional group^8^. A similar observation of lifted Al valence state is also observed in aluminum implanted in polyethylene terephthalate (PET) system to form Al-O-C species with PET^6^. Moreover, the carboxyl oxygen and the carboxyl carbon in PMMA are reactive^7^ with metal clusters and this pair of peaks indeed disappear without PMMA (Figure S6). Thus, the low valence state Mo ingredients indicate some interactions between PMMA and Mo clusters.


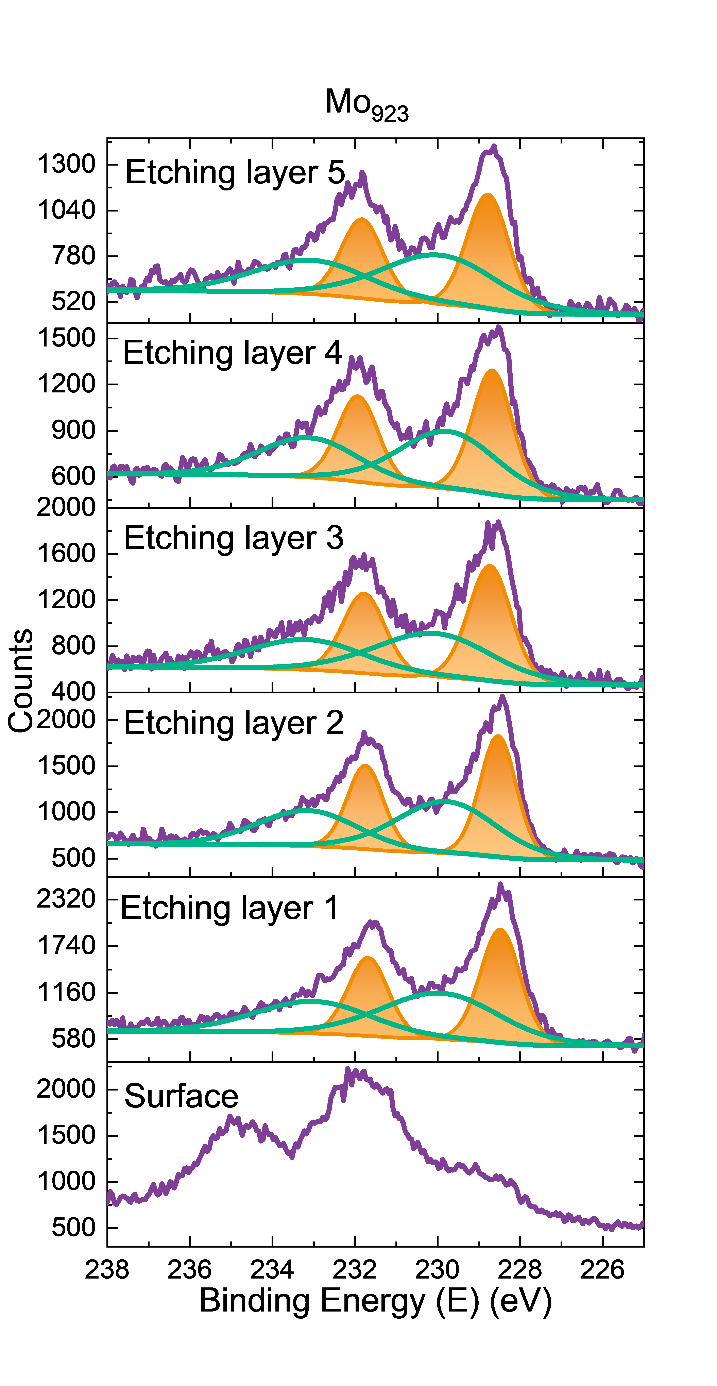


Figure S5: XPS spectra of Mo_923_ clusters packaged in PMMA after 5 times etching.


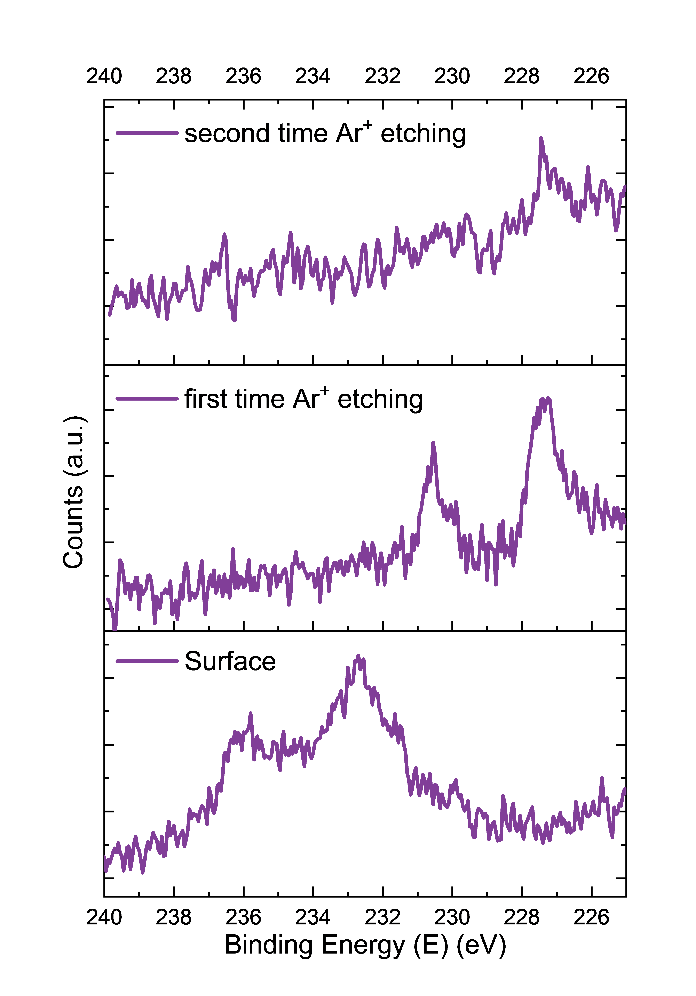


Figure S6: XPS spectra of Mo_923_ clusters directly deposited on silicon slice (sample 2). Compared with the results from Mo_923_ clusters packaged in PMMA in Figure S6 (sample 1), obvious differences appear. Firstly, for the surface layer, almost no shoulder located from 228 to 230 eV in XPS spectra appears in sample 2. Secondly, in the first etched layer of sample 2, Mo 3d XPS peaks show much shaper slope where no green-colored peaks corresponding to cluster-PMMA interaction can be resolved. Thirdly, after 2 times etching of sample 2, Mo 3d XPS signal decays almost to zero, while Mo 3d XPS signal of sample 1 where clusters distributed in PMMA remains strong even after 5 times etching (see results in Figure S6).


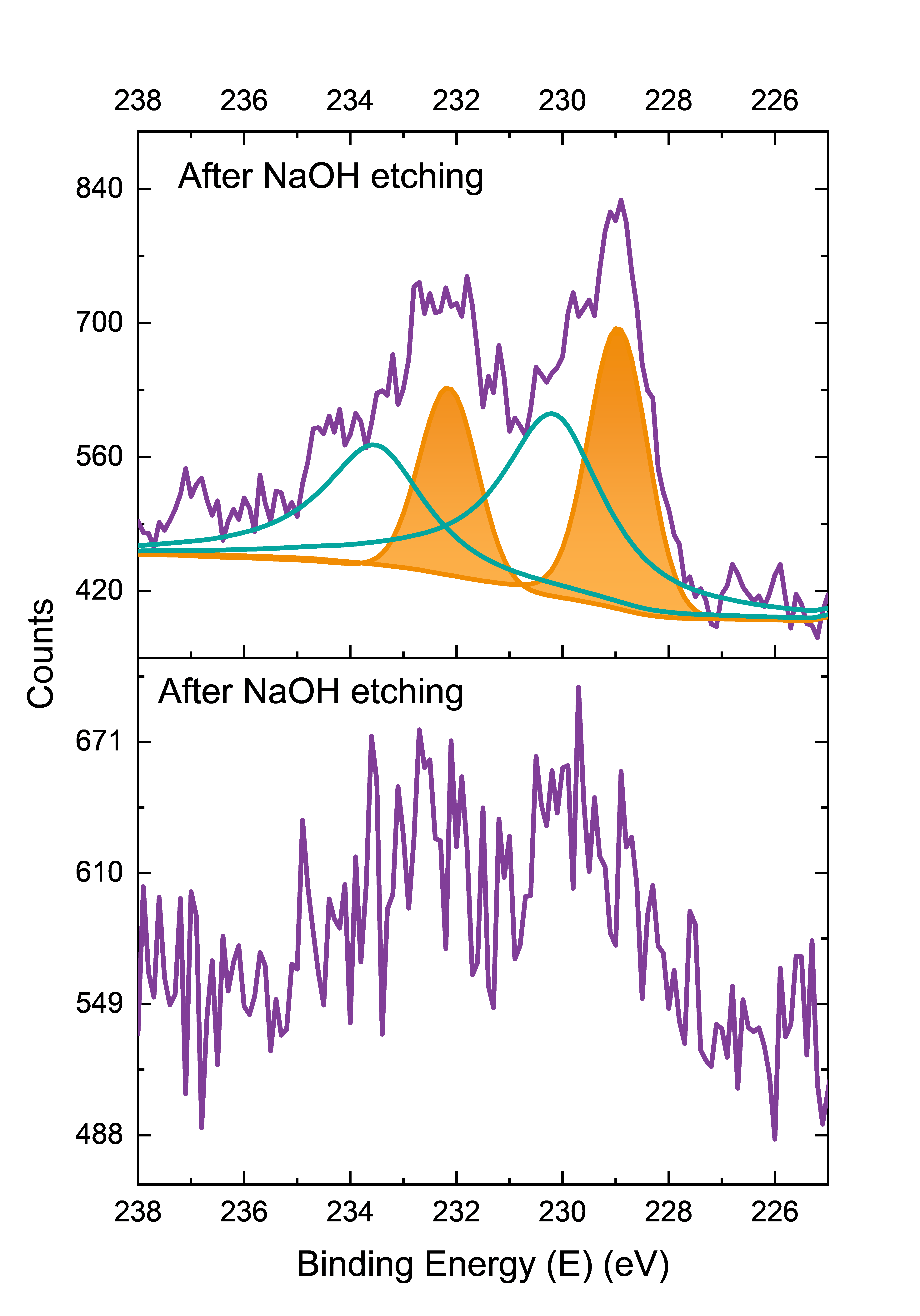


Figure S7: Two samples of XPS spectra from surface of Mo_923_-PMMA film after NaOH etching. After NaOH etching, high-valence state of Mo disappears (~232 eV). The result of XPS indicates the successful removal of surface residual oxidation clusters.


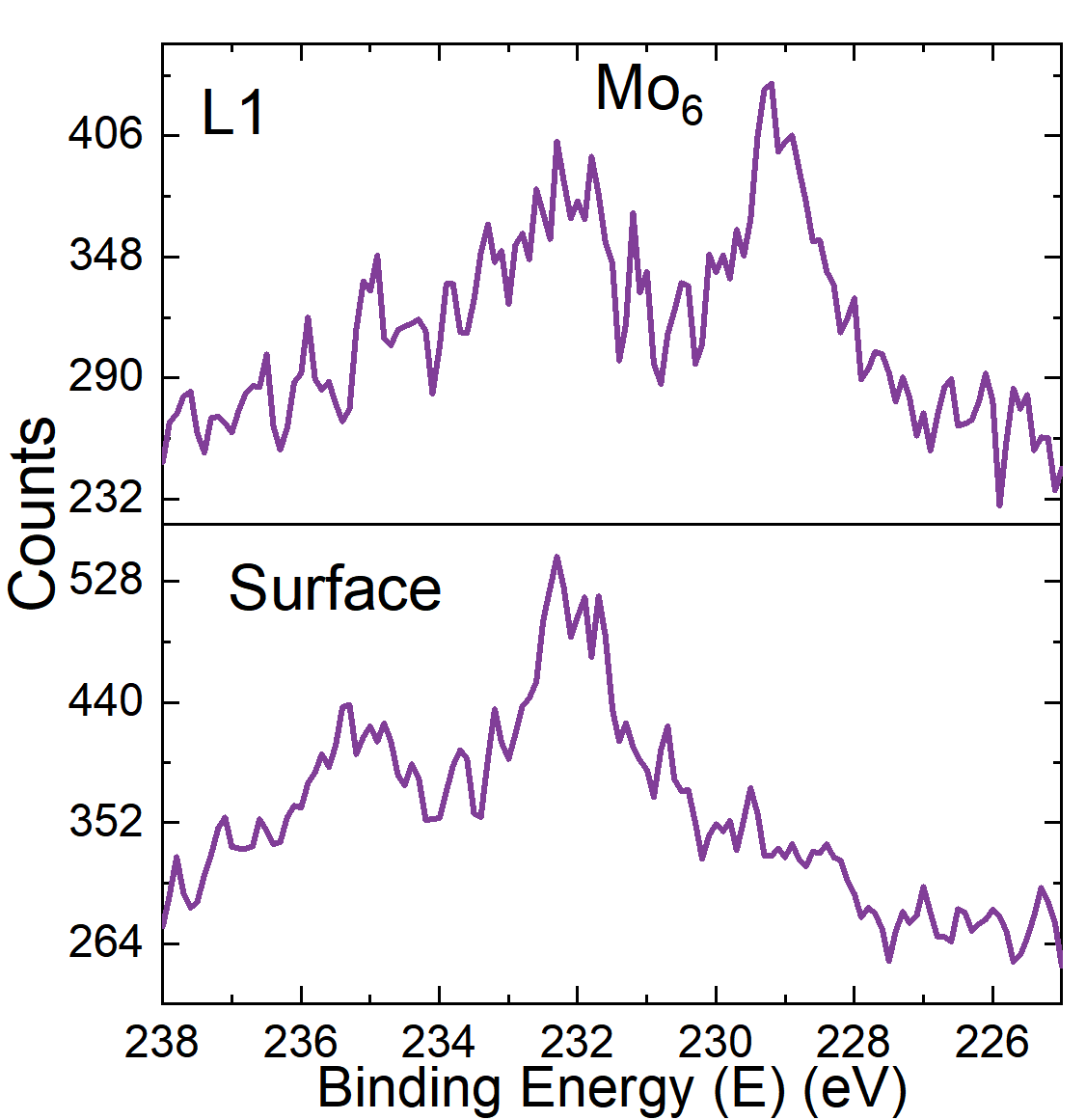


Figure S8: XPS spectra of Mo_6_ on PMMA surface (bottom) and packaged inside PMMA (top).


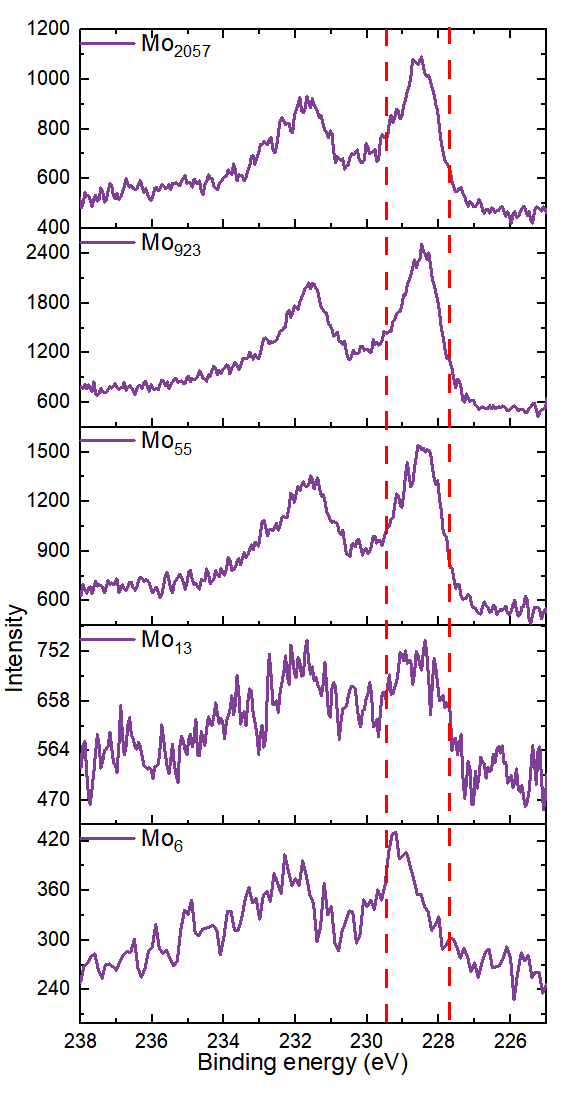


Figure S9: Overall XPS spectra of Mo clusters inside PMMA. An obvious blue shift of Mo 3d_5/2_ are found in Mo_6_ with a smaller slope.

Figure S10: (a) Adsorption sites of the MMA molecule on the Mo_6_ cluster, including the top site (A), bridge site (B), and hollow site (C). (b) The most stable configuration of the adsorbed complex. The relaxed structure indicates that the top site is the most stable, with an adsorption energy of 1.18 eV.


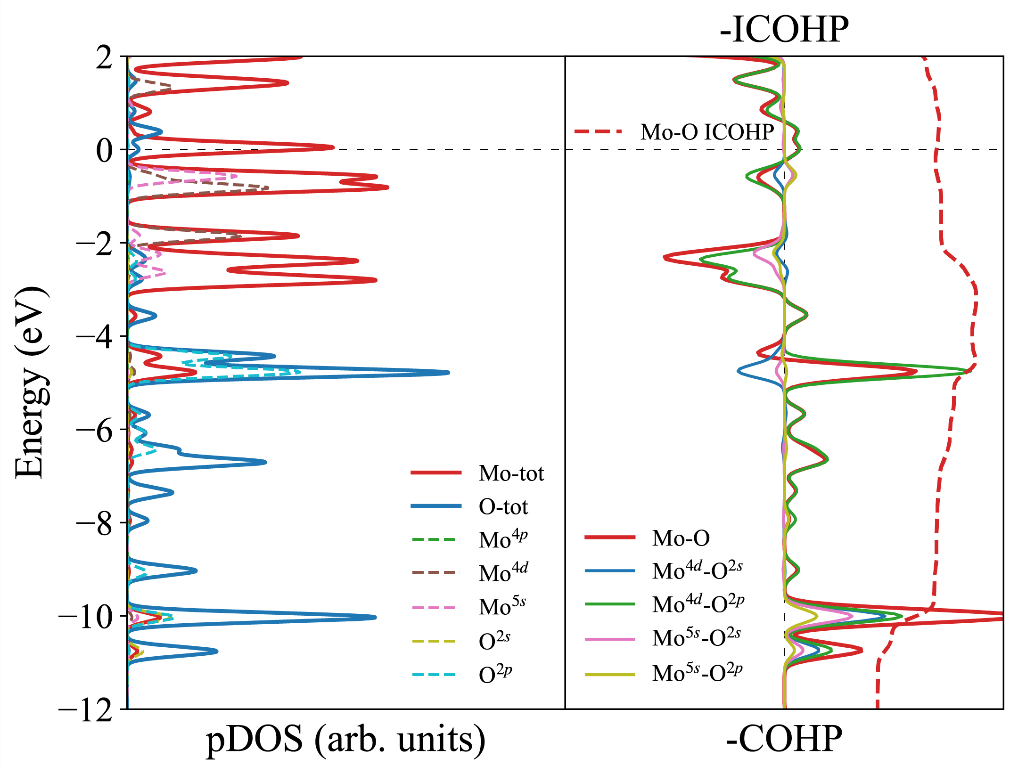


Figure S11: the projected Density of States (pDOS) and (Integral) Crystal Orbital Hamilton Population ((I)COHP) analysis of the Mo_6_-MMA complex, with the Fermi level set at 0 eV.


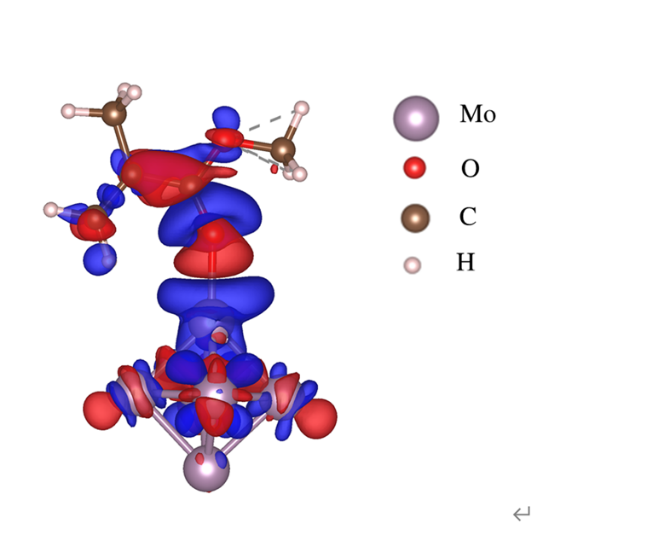


Figure S12: Charge transfer between the Mo_6_ cluster and the MMA molecule, where blue regions represent electron depletion and red regions indicate electron accumulation. (IsoSurface = 0.001).


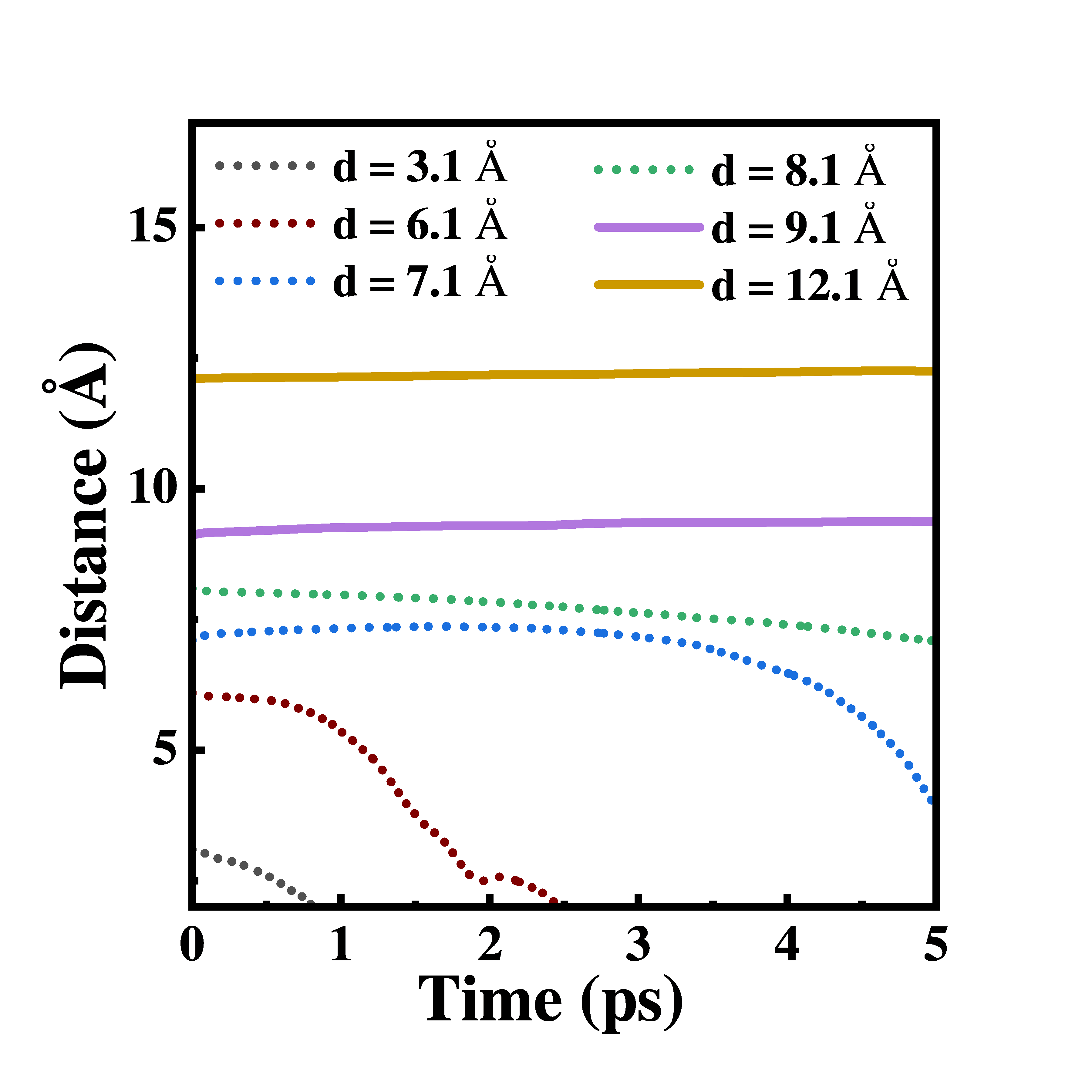


Figure S13: Dynamics of two Mo_6_ clusters initiated with varying nearest cluster distances.

To examine the interactions between two Mo_6_ clusters, we employed ab initio molecular dynamics simulations. At 50 °C, the interaction distance between the Mo_6_ nanoclusters is approximately 9.1 Å, as illustrated in Fig. S13.


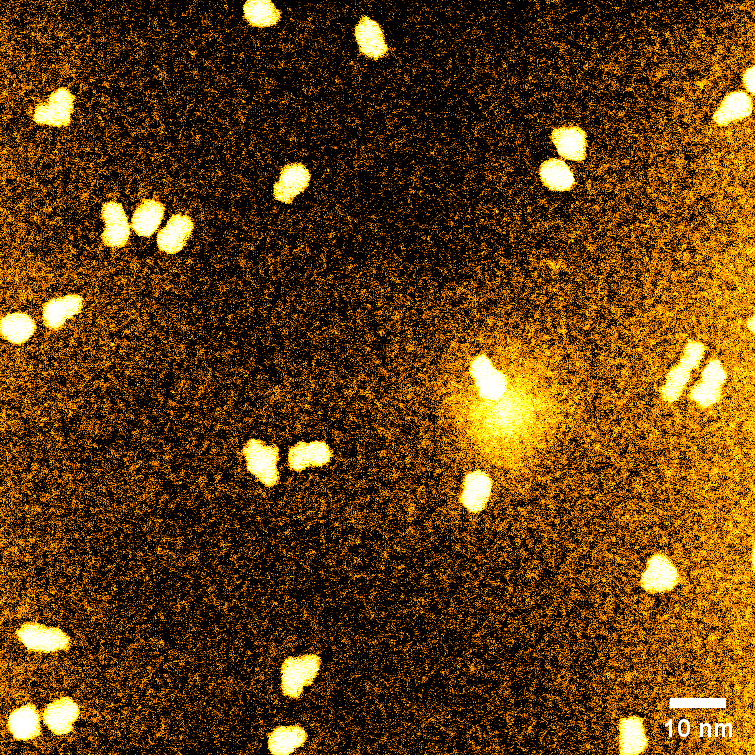


Figure S14: Co-deposited Mo_923_ clusters under 100 °C on carbon films suffer serious agglomerate. Many clusters are in strip shape with obvious larger size, indicating the aggregative growth^9^ among them.


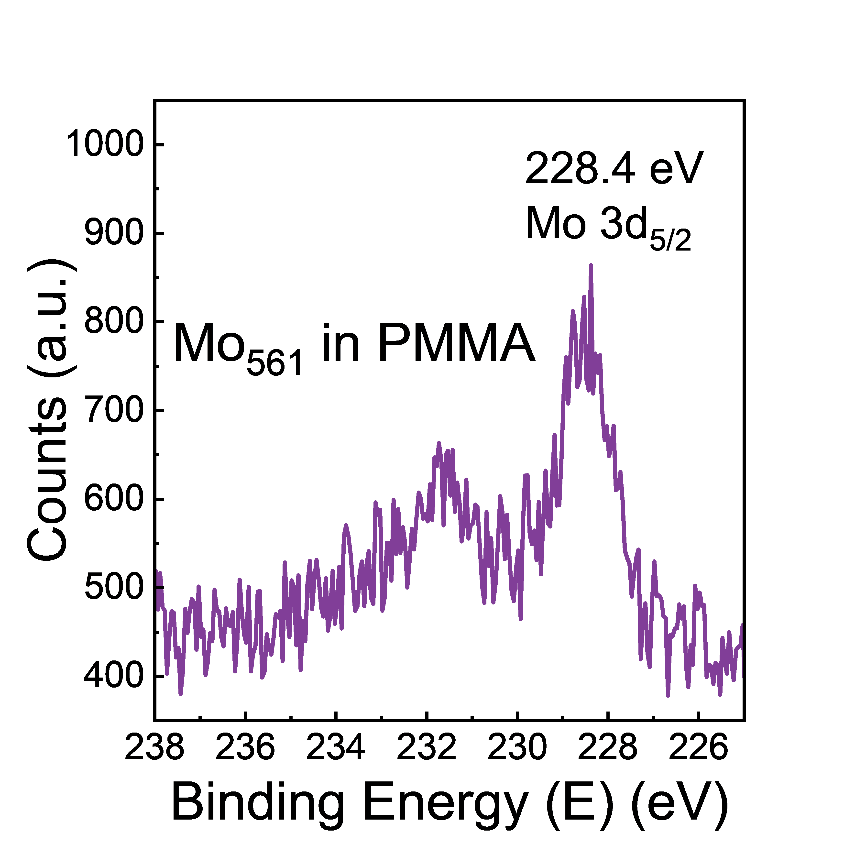


Figure S15: Mo 3d XPS spectra of packaged Mo_561_ in PMMA. It is obvious that Mo_561_ clusters are not oxidized as well. After acquiring XPS, this Mo-PMMA film was dissolved in anisole and redropped on a carbon film for STEM.


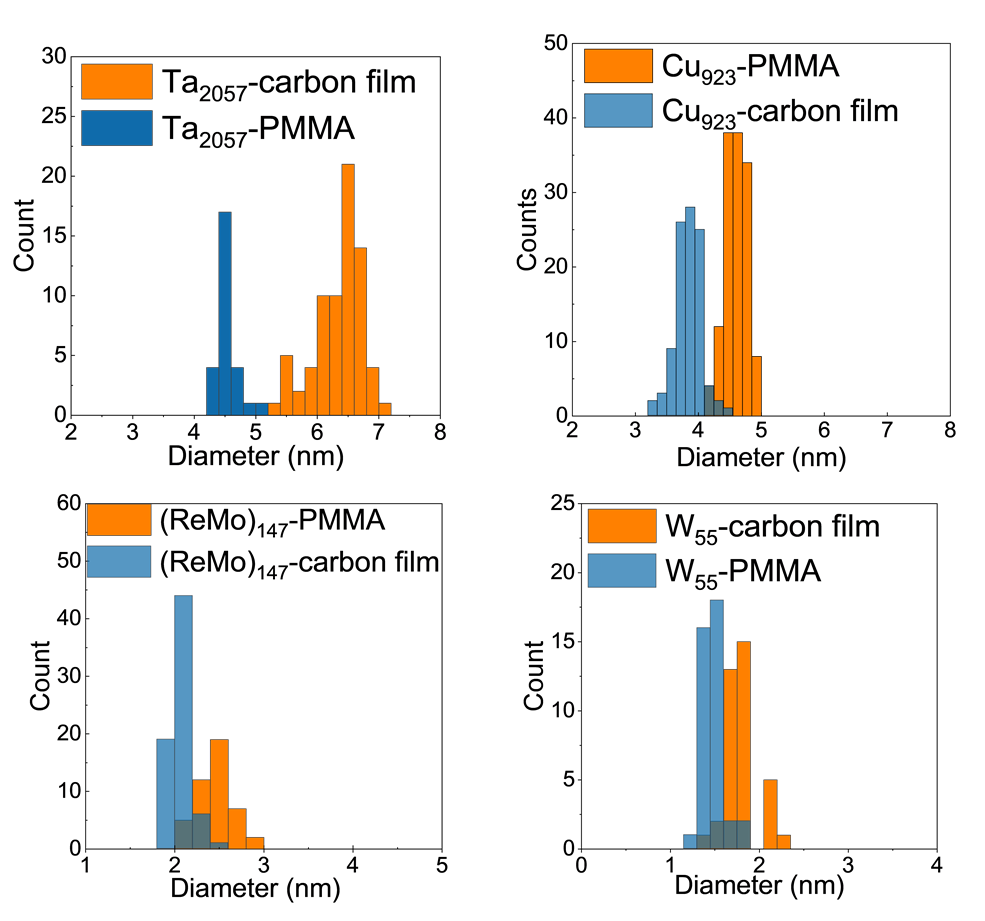


Figure S16: The comparison of air exposed and packaged clusters inside PMMA related to clusters in Figure 4.


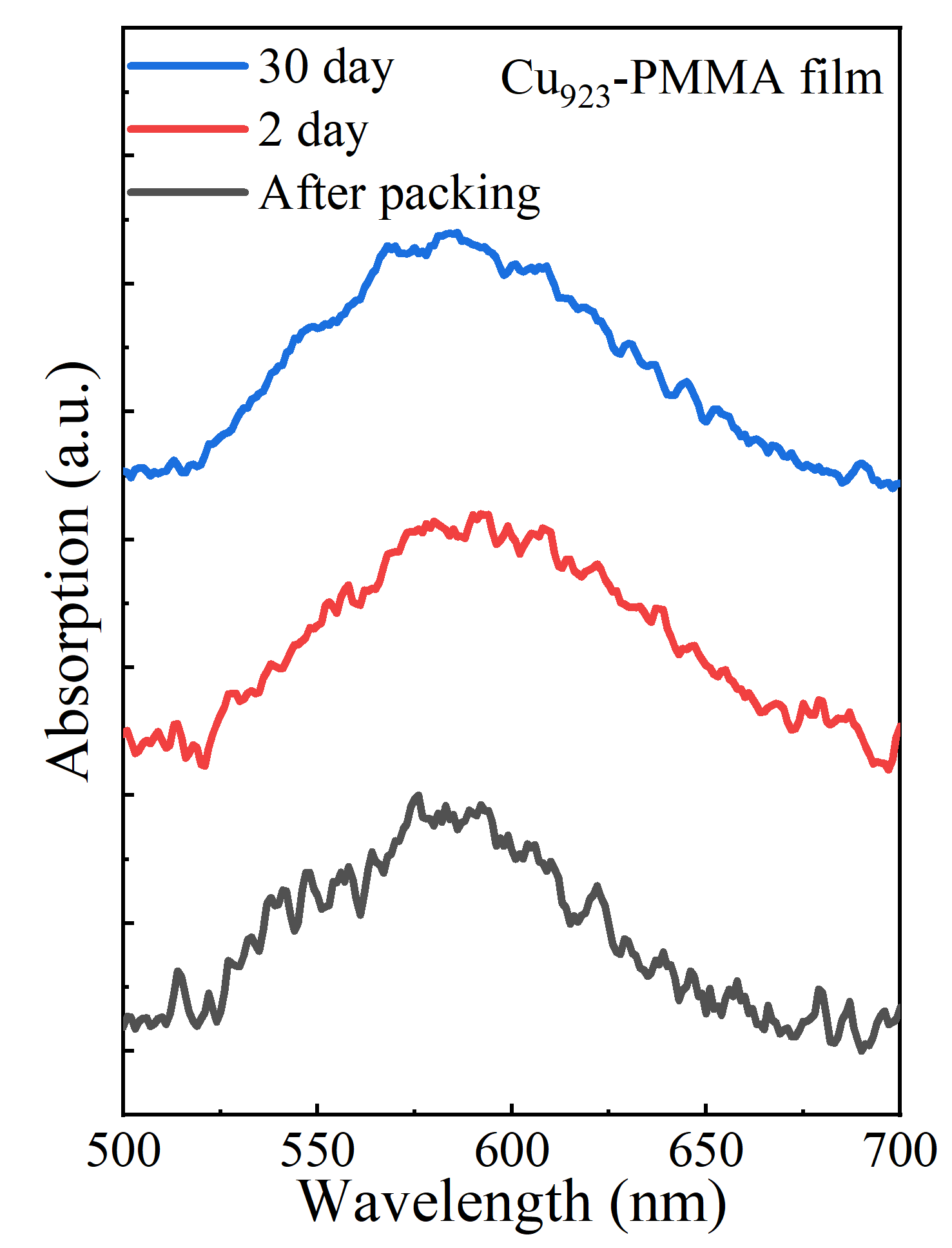


Figure S17: Normalized UV-vis spectra of packaged Cu_923_ clusters in PMMA. An obvious absorption peak located at ~585 nm is found which corresponds to Cu cluster plasmonic absorption10. No peak movement appears even after a month exposure in air. Compared to recent works where an absorption peak shift to over 600 nm happens when Cu nanoparticles are treated with ozone^11^ or exposed in air,^12^ It is clear that packaged Cu_923_ clusters in PMMA show long-term stable optical property.


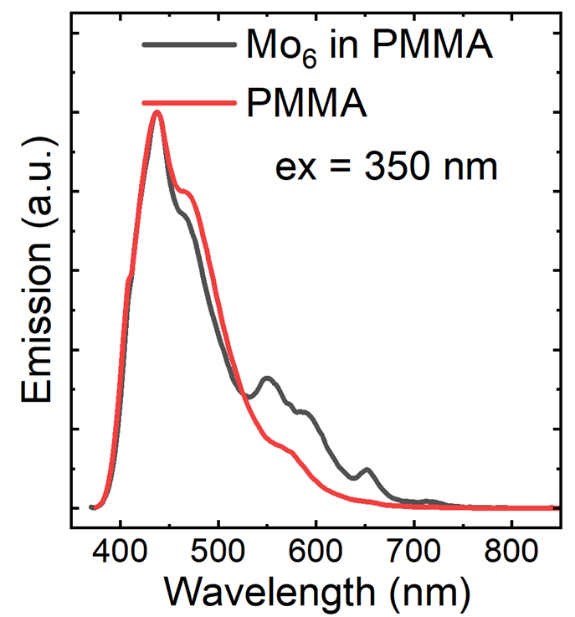


Figure S18: Normalized Photoluminescence spectra of packaged Mo_6_ clusters in PMMA (black) and pure PMMA (red) under 350 nm excitation.

Supporting Tables

Table S1. ICOHP results of Mo_13_-MMA complex. Bonding states form between Mo^4d^-O^2s^ and Mo^4d^-O^2p^.

| Mo_13_-MMA | -ICOHP |
| --- | --- |
| Mo^4d^-O^2s^ | 0.691 |
| Mo^4d^-O^2p^ | 1.16946 |

Table S2. Mulliken and Löwdin charge analysis (MCA and LCA) of the Mo_6_-MMA complex. In both MCA and LCA, positive values indicate electron depletion, while negative values indicate electron accumulation.

| Molecule | MCA (e) | LCA (e) |
| --- | --- | --- |
| Mo_6_ | 0.23 | 0.2 |
| MMA | -0.23 | -0.2 |

Supporting Methods

**LAMMPS simulation**

Classic molecular dynamics (MD) simulations are performed to explore the impacting behaviors of polymethyl methacrylate (PMMA) by metallic nanocluster from molecular level. First, a linear PMMA polymer chain composed of 100 monomers is generated. Then, 1200 PMMA chains are randomly placed in a MD box with dimensions of 300 × 300 × 900 Å^3^. Periodic boundary conditions (PBCs) are imposed in the orthogonal directions with small edge length, while non-PBC is applied in the orthogonal direction with the large edge length to mimic large-area PMMA substrate. Here, a coarse-grained model is employed to describe the linear PMMA polymer^13^. A common 12-6 Lennard-Jones potential is utilized to describe the interactions between unlike particles in the PMMA systems, as well as those between impacting metallic particles and PMMA particles.

Before conducting impacting MD simulations, as-generated PMMA sample is structurally relaxed with 100 ns at 323 K under NPT (constant number of particles, constant pressure, and constant temperature) ensemble, in which confining pressure of 0.1 GPa is applied in the orthogonal directions with small edge length, while non-confining pressure is applied in the other orthogonal direction. However, a wall-boundary condition is applied to prevent the overflow of PMMA during the MD relaxation process. As the MD relaxation is accomplished, a gold nanocluster composed of 309 atoms with ino-decahedral structure is placed on the top of as-relaxed PMMA polymer with distance of 10 nm. The choice of gold is to avoid any possible reaction between PMMA and clusters^1^ to purely focus on the implantation process. Finally, the gold nanocluster with kinetic energy of 100 eV moves downward the PMMA substrate, in which the impacting MD simulation is performed under micro-canonical ensemble (NVE). To avoid the global movement of PMMA substrate, a thickness layer of 10 nm in the bottom of PMMA substrate is frozen during the impacting MD simulation. The velocity-Verlet integration algorithm fs is utilized to integrate the Newton’s motions in the MD calculations. All MD calculations are implemented using the Large-scale Atomic-Molecular Massively Parallel Simulator (LAMMPS) software package^14^.

**Oxidation of Ta_561_ before implantation**

The oxidized process for Ta clusters before implantation is carried out through mixing O_2_ in sputtering Argon gas during the sputtering process within the condensation chamber in our home-made cluster source. The use of Ta instead of Mo is due to the quench of Mo cluster generation if we pump enough oxygen to the sputtering & condensation chamber and almost no Mo clusters can acquire. However, for Ta clusters with lower oxidation potential, the oxidation is easy to achieve when only a little bit of oxygen is pumped. The oxidized clusters are further packaged into PMMA in the deposition chamber under the pressure lower than 10^-5^ pa.

**FTIR and ellipsometer investigation of NaOH etched PMMA film**

FTIR spectra of PMMA on double-polished intrinsic silicon as well as in KBr was carried out on an IRAffinity-1S Fourier Infrared Spectrometer in transmission mode ranging from 500 to 4000 cm^-1^. Refractive index n and extinction coefficient k of PMMA are acquired by an ellipsometer modeled HORIBA France SAS.

**UV-vis absorption of Cu_923_-PMMA optical film**

The Cu_923_-PMMA optical film was made up in the following step: 1) an extra PVA film was spin coated on silicon slice before PMMA spin-coating; 2) the acquired PMMA-PVA-Silicon slice was used for cluster implantation; 3) after implantation, deionized water was used to dissolve PVA film, and Cu_923_-PMMA films floated on the water. 4) after using quartz flakes to scoop up the film, 0.1 mol/L HCl was used to wash away Cu oxide exposed on PMMA surface. UV-vis spectra of Cu_923_ in PMMA films was carried out on a METASH UV-6000PC ultraviolet and visible spectrophotometer in a transmission mode. It is worth noting that this low concentration HCl at room temperature do no destruction on PMMA film^15^ but remove Cu oxides.

**PL spectra of Mo_6_-PMMA film**

Photoluminescence spectra of Mo_6_-PMMA film was acquired on Hitachi F-7000 Steady State and Transient State Fluorescence Spectrometer. The cluster-PMMA films was transferred to a intrinsic silicon wafer with 400 μm thickness for spectra acquirement.

Supporting References

1. Tannenbaum R*, et al.* FTIR Characterization of the Reactive Interface of Cobalt Oxide Nanoparticles Embedded in Polymeric Matrices. *The Journal of Physical Chemistry B* **110**, 2227-2232 (2006).

2. Çaykara T, Güven O. UV degradation of poly(methyl methacrylate) and its vinyltriethoxysilane containing copolymers. *Polymer Degradation and Stability* **65**, 225-229 (1999).

3. Chase MW, Jr., Curnutt JL, Downey JR, Jr., McDonald RA, Syverud AN, Valenzuela EA. JANAF Thermochemical Tables, 1982 Supplement. *Journal of Physical and Chemical Reference Data* **11**, 695-940 (1982).

4. Eckhard JF, Neuwirth D, Tschurl M, Heiz U. From oxidative degradation to direct oxidation: size regimes in the consecutive reaction of cationic tantalum clusters with dioxygen. *Phys Chem Chem Phys* **19**, 10863-10869 (2017).

5. Xie FY*, et al.* XPS studies on surface reduction of tungsten oxide nanowire film by Ar+ bombardment. *Journal of Electron Spectroscopy and Related Phenomena* **185**, 112-118 (2012).

6. Bou M, Martin JM, Le Mogne T, Vovelle L. Chemistry of the interface between aluminium and polyethyleneterephthalate by XPS. *Applied Surface Science* **47**, 149-161 (1991).

7. Leadley SR, Watts JF. The Use of Monochromated XPS to Evaluate Acid-Base Interactions at the PMMA/Oxidised Metal Interface. *The Journal of Adhesion* **60**, 175-196 (1997).

8. Guo Z, Henry LL, Palshin V, Podlaha EJ. Synthesis of poly(methyl methacrylate) stabilized colloidal zero-valence metallic nanoparticles. *J Mater Chem* **16**, 1772-1777 (2006).

9. Van Hyning DL, Klemperer WG, Zukoski CF. Silver Nanoparticle Formation:  Predictions and Verification of the Aggregative Growth Model. *Langmuir* **17**, 3128-3135 (2001).

10. Kumar P, Mathpal MC, Swart HC. Multifunctional properties of plasmonic Cu nanoparticles embedded in a glass matrix and their thermodynamic behavior. *Journal of Alloys and Compounds* **747**, 530-542 (2018).

11. Popok VN. Formation and applications of polymer films with gas-phase aggregated nanoparticles: A brief review. *Thin Solid Films* **756**, (2022).

12. Popok VN, Hanif M, Ceynowa FA, Fojan P. Immersion of low-energy deposited metal clusters into poly(methyl methacrylate). *Nuclear Instruments and Methods in Physics Research Section B: Beam Interactions with Materials and Atoms* **409**, 91-95 (2017).

13. Zhang L-W, Ji W-M, Hu Y, Liew KM. Atomistic Insights into the Tunable Transition from Cavitation to Crazing in Diamond Nanothread-Reinforced Polymer Composites. *Research* **2020**, (2020).

14. Plimpton S. Fast Parallel Algorithms for Short-Range Molecular Dynamics. *Journal of Computational Physics* **117**, 1-19 (1995).

15. Ali U, Karim KJBA, Buang NA. A Review of the Properties and Applications of Poly (Methyl Methacrylate) (PMMA). *Polymer Reviews* **55**, 678-705 (2015).
